# Clinical, pathological and functional characterization of riboflavin-responsive neuropathy

**DOI:** 10.1093/brain/awx231

**Published:** 2017-09-26

**Authors:** Andreea Manole, Zane Jaunmuktane, Iain Hargreaves, Marthe H R Ludtmann, Vincenzo Salpietro, Oscar D Bello, Simon Pope, Amelie Pandraud, Alejandro Horga, Renata S Scalco, Abi Li, Balasubramaniem Ashokkumar, Charles M Lourenço, Simon Heales, Rita Horvath, Patrick F Chinnery, Camilo Toro, Andrew B Singleton, Thomas S Jacques, Andrey Y Abramov, Francesco Muntoni, Michael G Hanna, Mary M Reilly, Tamas Revesz, Dimitri M Kullmann, James E C Jepson, Henry Houlden

**Affiliations:** 1 Department of Molecular Neuroscience and Neurogenetics Laboratory, UCL Institute of Neurology, Queen Square, London, WC1N 3BG, UK; 2 MRC Centre for Neuromuscular Diseases, UCL Institute of Neurology, Queen Square, London, WC1N 3BG, UK; 3 Division of Neuropathology and Department of Neurodegenerative Disease, UCL Institute of Neurology, Queen Square, London, WC1N 3BG, UK; 4 Neurometabolic Unit, National Hospital for Neurology and Neurosurgery, London, WC1N 3BG, UK; 5 Department of Clinical and Experimental Epilepsy, UCL Institute of Neurology, Queen Square, London, WC1N 3BG, UK; 6 Reta Lila Weston Institute of Neurological Studies and Queen Square Brain Bank for Neurological Disorders, Queen Square, London WC1N 3BG, UK; 7 School of Biotechnology, Madurai Kamaraj University, Madurai 625021, India; 8 Departamento de Neurociências e Ciências do Comportamento, Faculdade de Medicina de Ribeirão Preto, Universidade de São Paulo (USP), Ribeirão Preto, SP, Brazil; 9 Chemical Pathology, Great Ormond Street Children’s Hospital, London, UK; 10 John Walton Muscular Dystrophy Research Centre, Institute of Genetic Medicine, Newcastle University, Central Parkway, Newcastle upon Tyne, UK; 11 Department of Clinical Neurosciences, School of Clinical Medicine, University of Cambridge, Cambridge CB2 0QQ, UK; 12 MRC Mitochondrial Biology Unit, Cambridge Biomedical Campus, Cambridge CB2 0QQ, UK; 13 NIH Undiagnosed Diseases Program, Common Fund, Office of the Director, Bethesda, MD, USA; 14 Laboratory of Neurogenetics, NIA/NIH, Bethesda, MD, USA; 15 Developmental Biology and Cancer Programme, UCL Great Ormond Street Institute of Child Health, 30 Guilford Street, London WC1N 1EH, UK; 16 The Dubowitz Neuromuscular Centre, UCL Great Ormond Street Institute of Child Health, 30 Guildford Street, London, WC1N 1EH, UK

**Keywords:** Brown-Vialetto-Van Laere syndrome, *SLC52A2*, *SLC52A3*, riboflavin

## Abstract

Brown-Vialetto-Van Laere syndrome represents a phenotypic spectrum of motor, sensory, and cranial nerve neuropathy, often with ataxia, optic atrophy and respiratory problems leading to ventilator-dependence. Loss-of-function mutations in two riboflavin transporter genes, *SLC52A2* and *SLC52A3*, have recently been linked to Brown-Vialetto-Van Laere syndrome. However, the genetic frequency, neuropathology and downstream consequences of riboflavin transporter mutations are unclear. By screening a large cohort of 132 patients with early-onset severe sensory, motor and cranial nerve neuropathy we confirmed the strong genetic link between riboflavin transporter mutations and Brown-Vialetto-Van Laere syndrome, identifying 22 pathogenic mutations in *SLC52A2* and *SLC52A3*, 14 of which were novel. Brain and spinal cord neuropathological examination of two cases with *SLC52A3* mutations showed classical symmetrical brainstem lesions resembling pathology seen in mitochondrial disease, including severe neuronal loss in the lower cranial nerve nuclei, anterior horns and corresponding nerves, atrophy of the spinothalamic and spinocerebellar tracts and posterior column–medial lemniscus pathways. Mitochondrial dysfunction has previously been implicated in an array of neurodegenerative disorders. Since riboflavin metabolites are critical components of the mitochondrial electron transport chain, we hypothesized that reduced riboflavin transport would result in impaired mitochondrial activity, and confirmed this using *in vitro* and *in vivo* models. Electron transport chain complex I and complex II activity were decreased in *SLC52A2* patient fibroblasts, while global knockdown of the single *Drosophila melanogaster* riboflavin transporter homologue revealed reduced levels of riboflavin, downstream metabolites, and electron transport chain complex I activity. This in turn led to abnormal mitochondrial membrane potential, respiratory chain activity and morphology. Riboflavin transporter knockdown in *Drosophila* also resulted in severely impaired locomotor activity and reduced lifespan, mirroring patient pathology, and these phenotypes could be partially rescued using a novel esterified derivative of riboflavin. Our findings expand the genetic, clinical and neuropathological features of Brown-Vialetto-Van Laere syndrome, implicate mitochondrial dysfunction as a downstream consequence of riboflavin transporter gene defects, and validate riboflavin esters as a potential therapeutic strategy.

## Teaser

Riboflavin metabolites are critical components of the mitochondrial electron transport chain. Manole *et al.* describe the genetics, phenotypes and neuropathology of a large patient series with riboflavin-responsive neuropathy. Using *in-vitro* and *in-vivo* models, they reveal mitochondrial dysfunction in the disorder, and validate riboflavin esters as a potential therapeutic strategy.

## Introduction

Brown-Vialetto-Van Laere (BVVL) syndrome is an autosomal recessive neurological disorder first described by Brown in 1894 and later by Vialetto and Van Laere (Brown, 1894; Vialetto, 1936; Van Laere, 1966). Affected patients mostly present with neuropathy, bilateral sensorineural deafness, bulbar palsy and respiratory compromise. Other cranial nerve palsies, optic atrophy, upper and lower motor neuron involvement and ataxia can occur particularly as disease progresses, mimicking conditions such as amyotrophic lateral sclerosis (ALS), Madras motor neuron disease and Nathalie syndrome (Anand *et al.*, 2012; Manole *et al.*, 2014). Deafness is the most common sign of this condition, with most affected individuals exhibiting hearing loss during the disease course. The time between the onset of deafness and the development of other manifestations varies but is usually in early childhood (Manole and Houlden, 2015).

Previous work has revealed strong links between mutations in two genes (*SLC52A2* and *SLC52A3*) and BVVL syndrome, both of which encode riboflavin transporters (Green *et al.*, 2010; Johnson *et al.*, 2012; Foley *et al.*, 2014). The role of another riboflavin transporter-encoding gene, *SLC52A1*, in BVVL syndrome pathogenicity is still uncertain, as it was found to be defective in only one case (Ho *et al.*, 2011). *SLC52A2* and *SLC52A3* mutations include missense, nonsense, frame-shift, and splice-site alterations, but uniformly result in loss-of-function through reduced riboflavin transporter expression and/or riboflavin uptake (Foley *et al.*, 2014; Udhayabanu *et al.*, 2016).

Riboflavin (7,8-dimethyl-10-ribityl-isoalloxazine; vitamin B_2_) is a water-soluble compound and acts as a precursor for flavin mononucleotide (FMN) and flavin adenine dinucleotide (FAD). Both FMN and FAD function in biological redox reactions such as in the mitochondrial electron transport chain (ETC) (Powers, 2003). Since riboflavin cannot be synthesized by mammals *de novo*, riboflavin transporters are indispensable for normal cellular metabolism, suggesting that reduced intracellular riboflavin is a critical pathological mediator of BVVL syndrome. Indeed, although infants with early-onset riboflavin transporter deficiency rapidly become ventilator-dependent and usually die in the first year of life, treatment with high-dose riboflavin supplementation partially ameliorates the progression of this neurodegenerative condition, particularly if initiated soon after the onset of symptoms (Foley *et al.*, 2014). In a recent paper by Rizzo *et al.* (2017), motor neurons from induced pluripotent stem cells (iPSCs) were derived from two patients with BVVL syndrome carrying either *SLC52A2* or *SLC52A3* mutations. They found a reduction in axon elongation coupled with perturbations in neurofilament composition and reduced autophagic/mitophagic flux. However, despite these findings the pathophysiological mechanisms underlying motor neuron degeneration in BVVL syndrome are still unclear.

Motor neurons are thought to be uniquely susceptible to impaired energy metabolism because of their high metabolic rate and axonal length (Cozzolino and Carri, 2012). Furthermore, mitochondrial perturbations causing alteration of the ETC and increased oxidative stress are thought to be involved in the pathomechanisms of neurodegeneration in some motor neuron diseases such as ALS or spinal muscular atrophy (Johnson *et al.*, 2010; Cozzolino and Carri, 2012; Bartolome *et al.*, 2013). Given the critical role of riboflavin in the generation of substrates used for the ETC, we hypothesized that reduced riboflavin transport results in impaired ETC, which may in turn contribute to neurodegeneration.

Here, we expand the clinic-genetic spectrum of riboflavin transporter genes and then perform a series of experiments to identify the underlying effects of loss of riboflavin transporter function on neuronal integrity and mitochondrial function. We review clinical case histories and undertake pathological evaluation of brain and spinal cord of two patients with confirmed *SLC52A3* mutations who presented either in infancy or in later childhood. Finally, we investigate the *in vitro* cellular effects of *SLC52A2* mutations on metabolism and ETC function and also the *in vivo* consequences of loss of the *SLC52A2*/*SLC52A3* homologue in *Drosophila melanogaster*, and test whether these can be mitigated by supplementation with a riboflavin derivative.

## Materials and methods

### Study subjects

Patients were enrolled and informed consent from the patient and/or their parental guardian was obtained. DNA was collected from a total of 132 suspected cases (probands and their relatives) presenting with cranial neuropathies and sensorimotor neuropathy with or without respiratory insufficiency. Patients’ DNA samples were collected at medical centres in England (including from patients originating from Pakistan, India, Saudi Arabia, Kuwait, Iran and Turkey) as well as from medical centres in Wales, Scotland, Northern Ireland, Ireland, France, Belgium, the Netherlands, Greece, Malta, Russia, Lebanon, Iceland, Australia and the USA, following the announcement of an on-going molecular study at the UCL Institute of Neurology, University College London (National Hospital for Neurology and Neurosurgery, Queen Square, London) of patients presenting with this phenotype. This study was ethically approved by the UCL/University College London Hospital Joint Research Office (99/N103), and written informed consent to perform a skin biopsy and fibroblasts was obtained as appropriate.

#### PCR and Sanger sequencing

Primer sequences, PCR and Sanger sequencing conditions for *SLC52A1, SLC52A2* and *SLC52A3* were as in Foley *et al.* (2014). Segregation of pathogenic variants was also assessed. Where one heterozygous mutation was identified, deletions in the other allele were investigated by array CGH (comparative genomic hybridization) but no deletions or insertions were identified. Mutation positions are based on NCBI reference sequences for complementary DNA. *SLC52A2* mutation positions are based on sequences NM_024531.4 for the nucleotide sequence and NP_078807.1 for the protein sequence. *SLC52A3* mutation positions are based on sequences NM_033409.3 for the nucleotide sequence, and NP_212134.3 for the protein sequence.

#### Neuropathological evaluation

Formalin-fixed, paraffin-embedded brain and spinal cord tissue were available from two patients (Patients AM2 and AM4). The paraffin sections were cut at 4 µm thickness, mounted on glass slides and stained with routine haematoxylin and eosin, and Luxol® fast blue/cresyl violet histochemical stains. Sections were examined by immunohistochemistry with the following antibodies: rabbit anti-glial fibrillary acid protein (GFAP) (polyclonal, 1:2500, Dako), mouse anti-phosphorylated neurofilaments (clone SMI31, 1:5000, Sternberg), mouse anti-myelin basic protein (clone SMI94, 1:2000, Sternberg), rabbit anti-ubiquitin (polyclonal, 1:1200, Dako), mouse anti-p62 (3/P62LCK Ligand, 1:100, BD Transduction), mouse anti-α-synuclein (clone KM51, 1:50, Leica/Novocastra), mouse anti-amyloid-β (clone 6F3D, 1:100, Dako), mouse anti-hyperphosphorlyated tau (clone AT8, 1:1200, Innogenetics), mouse anti-TDP43 (clone 2E2-D3, 1:3000, Abova), mouse anti-CD68 (clone PG-M1, 1:100, Dako), mouse anti-CD3 (LN10, 1:100, Leica/Novocastra), mouse anti-CD20 (clone 7D1, 1:200, Dako). Immunohistochemistry was carried out either manually or on a BOND-MAX autostainer (Leica Microsystems) using 3,3′-diaminobenzidine as chromogen. Negative controls were treated identically except that the primary antibody was omitted. Appropriate positive controls were used for all immunohistochemical studies.

#### *SLC52A2* patient fibroblasts

For further details of the generation of human fibroblast cultures and cell culture, determination of flavin status in fibroblasts, and assessment of ETC complex I, II, and citrate synthase activities in fibroblasts see the Supplementary material.

### *Drosophila* studies

For details of semi-quantitative RT-PCR of whole flies, measurement of mitochondrial membrane potential, isolation of *Drosophila* mitochondria and measurement of mitochondrial respiration, and immunohistochemistry of the *Drosophila* larval neuromuscular junction, see Supplementary material.

#### Stocks and culture conditions

Fly strains were obtained from the Bloomington *Drosophila* Stock Center (Indiana, USA) and Vienna *Drosophila* Resource Center (Austria). All transgenic insertions used in this study were outcrossed at least five times into an isogenic (iso31) background. These were: *cg11576* UAS-RNAi (VDRC 7578, Dietzl *et al.*, 2007; and HMC04813, Perkins *et al.*, 2015), and *daughterless*-GAL4 (Bloomington stock 55850). Flies were reared at 25°C on standard fly food consisting of corn meal, yeast, sucrose, glucose, wheat germ, soya flour, nipagin and propionic acid. For experiments with compound supplementation, 0.1 mg/ml riboflavin (Sigma) or 0.1 mg/ml riboflavin-5'-lauric acid monoester (RLAM) was added to the food. Flies were kept under 12-h light: 12-h dark cycles.

#### Quantification of flavins, ETC complex I, II, II/III redox activities and citrate synthase assay

Riboflavin, FMN and FAD content, and ETC complex I, II redox activity and citrate synthase activities were measured in flies (∼10/genotype) as described above for patient fibroblasts. Prior to analysis all samples were subjected to three cycles of freeze/thawing to lyse membranes. A Lowry assay was used to normalize for protein concentration (Frolund *et al.*, 1995). Complex II/III activity was determined at 30°C using the method of King (1967), which followed the succinate-dependent antimycin A sensitive reduction of cytochrome *c* at 550 nm.

#### Electron microscopy of fly brains

Flies were decapitated and the heads were perfused with 3% glutaraldehyde in 0.1 M sodium cacodylate buffer and 5 mM CaCl_2_ overnight. Brains were then treated with 1% osmium tetroxide for 3 h at 4°C and embedded in Araldite® CY212 resin. Thin sections were stained with toluidine blue and the brain visualized with the light microscope. Ultrathin sections (70 µm) were stained with lead citrate and uranyl acetate and digital images taken on a Philips CM10 TEM with MegaView G3 digital imaging system with Radius Software (Olympus). Mitochondrial morphology was measured blind to genotype.

#### Larval behaviour

Larval locomotion was tested by placing individual third instar larvae in the centre of petri dishes (8.5 cm diameter, 1.4 cm height) coated with 10 ml of 4% agar. On average, 30 larvae were tested per strain. The number of grid squares (1 cm) entered per min by the larva was measured per each genotype.

#### Adult behaviour

Adult flies were kept as groups of males and females in a 12 h:12 h light-dark cycle at 25°C for 1 day prior to testing. Single virgin females (∼1 day old) were loaded into glass tubes (with 2% agar and 4% sucrose food) and monitored using the *Drosophila* Activity Monitoring System (Trikinetics) in 12 h:12 h light-dark conditions at 25°C with an approximate intensity of 700–1000 lx during the light condition. For experiments involving supplementation, riboflavin or RLAM was added to the above food. Fly activities were deduced from the number of times flies broke beams of infrared light passing through the middle of the tube. Locomotor activity was recorded in 30-min bins and an analysis was performed on the second day after loading. Data were pooled from at least two independent experiments. The relative locomotor activities per 30-min bin for individual flies were averaged for each genotype and also the average locomotor activity per day was calculated. Locomotion graphs were generated using GraphPad Prism 6 and Microsoft Excel.

#### Life span

Adult female flies were collected from eclosion and transferred to fresh food tubes (10 flies/tube) with or without RLAM supplementation. Each day, death events were scored and viable flies were transferred to fresh tubes. Survival proportions were plotted as percentage of live flies against days. Approximately 100 flies were tested for each genotype.

### Statistics

Statistics were performed using GraphPad Prism 6. The significance between the variables was shown based on the *P*-value obtained (ns indicates *P* > 0.05, *P < 0.05, **P < 0.005, ***P < 0.0005). Data are presented as box plots illustrating 80% of the data distribution, together with the median and 10th, 25th, 75th and 90th percentiles unless otherwise stated.

## Results

### Clinical and genetic analysis of patients with BVVL syndrome

We screened 132 patients with phenotypes suggestive of BVVL syndrome and identified 20 patients (15%) with riboflavin transporter mutations. Genetic and clinical details of the 20 individuals carrying mutations in *SLC52A2* or *SLC52A3* are summarized in Table 1. Thirteen of the probands were males. Age of symptom onset was available for 15 patients (mean: 8.2 years; range: 7 months–21 years). First symptoms usually occurred during childhood (11 patients) and less frequently during teenage years (four patients) or adulthood (one patient), and were mostly secondary to cranial nerve involvement (Table 1). Only one patient presented with symptoms not related to cranial nerve involvement (limb weakness). Sensorineural hearing loss was both a common presenting symptom (eight patients) and a common clinical feature during follow-up (17 patients). Other frequent clinical features include optic atrophy (14 patients), weakness of facial and bulbar muscles (13 patients) and sensorimotor peripheral axonal neuropathy (16 patients). Limb weakness was more severe in the upper than in the lower limbs in nine patients. Ten patients developed some degree of respiratory involvement with three requiring assisted ventilation, and 10 patients had dysphagia and/or chewing difficulty, six of them requiring nasogastric tube or gastrostomy feeding.

**Table 1:** Clinical and genetic features of patients with Brown-Vialetto-Van Laere syndrome caused by mutations in *SLC52A2* and *SLC52A3* at the time of diagnosis

Patient	Mutation	ExAC allele count/ Hom	Sex	Ethnicity	First symptom	Age at first symptom	OA/HL	Sensorimotor neuropathy^b^	Distribution of weakness	Overall maximal motor function	Maximal motor function at the time of diagnosis	Respiratory function	Feeding	Age at genetic diagnosis
AP1	*SLC52A2* homozygous	4/0	F	English	Horizontal nystagmus	7 mo	OA	Yes	UL>LL	Crawling, could hold objects with pincer grip	Hypotonic, deteriorated arm function	Ventilator dependent	Gastrostnomy only	2.1 y^†^
	c.935T>C p.Leu312Pro													
AP2	*SLC52A2* compound heterozygous	[3/0][0/0]	F	Brazilian	Sensorineural hearing loss	Childhood	OA, HL	Yes	UL>LL	Can walk with walking devices and orthosis	Able to walk	Respiratory insufficiency	Not affected	27 y
	c.[383C>T];[1088C>T]													
	**p.[Ser128Leu][Pro363Leu]**													
AP3	*SLC52A3* heterozygous	1/0	M	English	UL weakness	19 y	HL	Yes	UL and LL	Wheelchair bound	Wheelchair bound	Ventilator dependent	Dysphagia	20 y
	c.1371C>G p.Phe457Leu													
AP4	*SLC52A3* heterozygous	2/0	M	Brazilian	Sensorineural hearing loss	n/a	HL	Yes	UL>LL	n/a	n/a	Not affected	n/a	35 y
	c.37G>A p.Gly13Arg													
AP5	*SLC52A3* heterozygous	2/0	M	Brazilian	Sensorineural hearing loss	n/a	HL	Yes	UL>LL	n/a	n/a	Not affected	n/a	35 y
	c.37G>A p.Gly13Arg													
AP6	*SLC52A2* compound heterozygous	[8/0][4/0]	M	English	Visual loss, optic atrophy	15 mo	OA, HL	Yes	UL>LL	Wheelchair bound	Holds pen in mouth to draw in tablet	Sleep apnoea	Oral diet	17 y
	c.[1016T>C];[c.935T>C]													
	p.[Leu339Pro][Leu312Pro]													
AP7	*SLC52A3* heterozygous	1/0	F	English	Sensorineural hearing loss	10 y	HL	Yes	UL>LL	n/a	n/a	Respiratory problems	n/a	12 y
	c.374C>A													
	**p.Thr125Asn**													
AP8	*SLC52A3* heterozygous	5/0	F	English	Partial right third nerve palsy	15 y	No	Yes	UL>LL	Wheelchair bound	Wheelchair bound	Not affected	Nasogastric tube	15 y
	c.403A>G													
	**p.Thr135Ala**													
AP9	*SLC52A3* heterozygous	10/0	M	English	Optic nerve atrophy	2 y	OA, HL	Yes	UL>LL	Unsteadiness, fatigue and falls	n/a	Respiratory problems	Difficulty chewing and swallowing	25 y
	c.58A>C													
	**p.Ile20Leu**													
AP10	*SLC52A3* compound heterozygous	[3/0][1/0]	M	English	n/a	n/a	OA, HL	Yes	n/a	Could walk a short distance with a stick	n/a	Not affected	n/a	n/a
	c.[106G>A];[c.1237T>C]													
	p.[Glu36Lys];[p.Val413Ala]^a^													
AM1	*SLC52A3* compound heterozygous	[0/0][0/0]	M	English	Sensorineural hearing loss	2 y	OA, HL	Yes	UL and LL	n/a	n/a	Breathing difficulty	Gastronomy only	22 y
	c.[354G>A][1074G>A]													
	**p.[Val118Met][splice defect]**													
AM2	*SLC52A3* compound heterozygous	[0/0][0/0]	M	English	Horizontal nystagmus, ptosis, neck weakness	8 mo	OA, n/a	Yes	UL>LL	Completely paralysed	Completely paralysed	Ventilator dependent	Gastronomy only	2 y^†^
	c.[1128-1129_insT][1294G>A]													
	**p.[Tyr376fs][Trp431X]**													
AM3	*SLC52A2* compound heterozygous	[1/0][9/0]	F	English	Sensorineural hearing loss	3 y	OA, HL	Yes	UL and LL	Unsteadiness, fatigue and falls	Unsteadiness, fatigue and falls	Breathing difficulty	Difficulty chewing and swallowing	54 y
	c.[231G>A];[c.865C>T]													
	**p.[Glu77Lys];[Ala288V]**													
AM4	*SLC52A3* compound heterozygous	[2/0][0/0]	M	English	Sensorineural hearing loss	8 y	OA, HL	Yes	LL	Mild postural tremor	Mild postural tremor	Compromised	Chewing difficult	19 y^†^
	c.[39G>A];[1255G>A]													
	p.[Gly13Arg]**[Gly418Asp]**													
AM5	*SLC52A2* homozygous	0/0	M	Arab	Nystagmus	2.5 y	OA, HL	Yes	LL>UL	Wheel chair bound	Crawls in bed using elbows	Not affected	Nasogastric tube	8 y
	c.1327T>C													
	**p.Cys443Arg**													
AM6	*SLC52A2* homozygous	0/0	F	Arab	Visual loss, optic atrophy	18 mo	OA, HL	Yes	UL and LL	Generalized hypotonia	n/a	Not checked	Nasograstic tube	4 y
	c.1327T>C **p.Cys443Arg**													
AM7	*SLC52A3* homozygous	1/0	M	Arab	Facial palsy and mild hearing lost	14 y	OA, HL	No	No	Some leg pain but can walk	Some leg pain but can walk	Not affected	Normal	17 y
	c.634C>T													
	**p.Arg212Cys**													
AM8	*SLC52A3* homozygous	1/0	M	Arab	Facial palsy and paralysed vocal cord	11 y	OA, HL	No	No	Some leg pain but can walk	Some leg pain but can walk	Not affected	Normal	15 y
	c.634C>T													
	**p.Arg212Cys**													
AM9	*SLC52A3* homozygous	1/0	M	Arab	No symptom	Not developed	No	No	No	Not checked	Not checked	Not affected	Normal	12 y
	c.634C>T													
	**p.Arg212Cys**													
AM10	*SLC52A3* homozygous	1/0	F	Arab	Facial palsy and mild hearing loss	14 y	OA, HL	No	No	Some leg pain but can walk	Some leg pain but can walk	Not affected	Normal	5 y
	c.634C>T													
	**p.Arg212Cys**													

Novel mutations are in bold.

^†^Deceased; F = female; HL = hearing loss; Hom = homozygous; LL = lower limb; M = male; mo = months; n/a = not available; OA = optic atrophy; UL = upper limb; y = years.

^a^In *cis* configuration.

^b^Based on nerve conduction studies.

Of the 22 mutations identified, eight were found in the *SLC52A2* locus and 14 in *SLC52A3*, of which five *SLC52A2* and nine *SLC52A3* variants were novel (Table 1). In contrast, no *SLC52A1* mutations were observed. None of the variants were found in the homozygous state in the ExAC database of over 100 000 controls, suggesting pathogenicity (Table 1). Consistent with this hypothesis, all except one mutation (p.Arg212Cys in *SLC52A3*) were predicted to be at least probably damaging by SIFT and PolyPhen-2 algorithms (scores ranging between 0.55 and 1). The mutations reside in transmembrane helices and in the intracellular and extracellular loops. Although three homozygous and seven compound heterozygous mutations were identified, five mutations (all in *SLC52A3*) were identified on only one allele. These heterozygous individuals did not differ substantially in phenotype including age of presentation from the rest of the cohort of mutation-positive cases.

There was no correlation between the nature of pathogenic variant and phenotype severity, although in the case of Patient AM2, nonsense mutations on both alleles resulted in a truncated protein, and this genotype correlated with rapid progression of symptoms and death at 2 years of age.

### Neuropathological analysis of patients with BVVL syndrome

To characterize in detail the neuropathological symptoms of BVVL syndrome, we undertook a comprehensive pathological examination of two patients carrying compound heterozygous *SLC52A3* mutations (Patients AM2 and AM4; Table 1). These patients represent two ends of the spectrum of severity of BVVL syndrome.

Patient AM2 had a normal birth at term. His motor skills were mildly delayed and he never acquired the ability to roll over completely front to back. He achieved the ability to sit with minimal support at age 7 months. From about 8 months of age he began to exhibit more clear signs of the condition such as ptosis and neck weakness. He was admitted at the age of 9 months for investigations but no diagnosis was made at that time. His condition quickly progressed to include respiratory muscle weakness, and ventilator dependence at the age of 1 year. He also developed severe weakness in his shoulder girdle areas and proximal upper limbs. Weakness subsequently developed in forearm muscles, distal lower limb muscles and thighs, trunk and face, and progressed to the point that he could only weakly move his eyelids and had very limited sideways movements of his eyes. Nerve conduction studies and EMG showed a severe neuropathy. He died at 2 years of age of respiratory failure.

**Figure 1:**
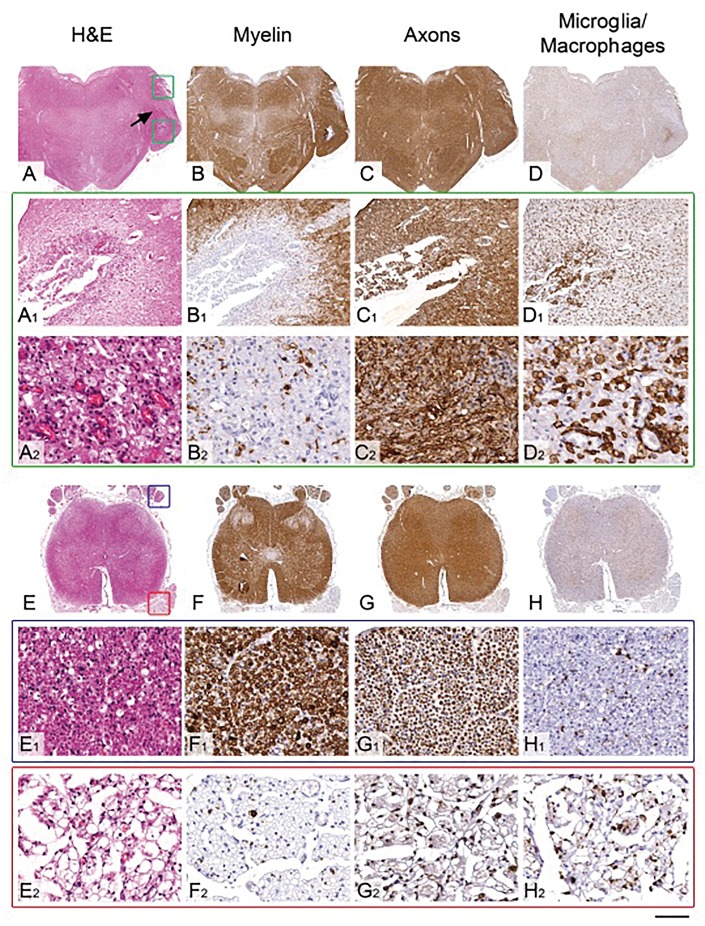
**Pathological features of the symmetrical brain stem lesions and the comparison of the spinal cord nerve root involvement in the Patient AM2.** (**A**–**D**) Low power views of the pons and (**A_1_**–**D_2_**) high power views of the pontine lesion indicated with green square boxes in **A**. (**A**–**A_2_**) The haematoxylin and eosin (H&E) stained section demonstrates sharply demarcated lesion surrounding the fifth cranial nerve (black arrow). High power view (**A_2_**) reveals frequent small calibre blood vessels and foamy macrophages within the lesion. (**B**–**B_2_**) Immunostaining for myelin with SMI94 antibody shows a near complete absence of myelin, whilst the axons (**C**–**C_2_**), demonstrated with SMI31 antibody, are well preserved within the lesion. (**D**–**D_2_**) Immunostaining for macrophages with CD68 antibody confirm a dense infiltrate of macrophage in the centre of the lesion. (**E**–**H**) Low power views of transverse sections of the sacral spinal cord. The posterior nerve roots are indicated with blue square box in **E** and on high power views in **E_1_**–**H_1_**. The anterior nerve roots are indicated with red square box in **E** and on high power views in **E_2_**–**H_2_**. (**E_1_**–**G_1_**) The posterior nerve roots are densely populated with myelinated fibres with (**H_1_**) minimal macrophage activity. (**E_2_**–**G_2_**) In the anterior nerve roots there is a severe loss of myelinated fibres and (**H_2_**) widespread infiltration of macrophages. Scale bar = 4 mm in **A**–**D**; 1.7 mm in **E–H**; 350 µm in **A_1_**–**D_1_**; 70 µm in **A_2_**–**D_2_**; 140 µm in **E_1_**–**H_1_** and **E_2_**–**H_2_**.

The cerebral cortex, hippocampus and cerebral white matter were unremarkable. The deep grey nuclei were not available for assessment. The neuronal density in the substantia nigra was normal for the patient’s age, but the third and fourth cranial nerve nuclei showed severe neuronal loss, gliosis and microglial activation (Supplementary Fig. 1). In the pons there were two symmetrical sharply demarcated lesions surrounding both fifth cranial nerves (Fig. 1A–D). In these lesions we observed prominent neovascularization, dense infiltration of macrophages and widespread myelin loss, with relative preservation of axons. The fifth cranial nerve was vacuolated and its nucleus showed severe neuronal loss and gliosis (Supplementary Fig. 1). In the medial lemniscus at the level of the midbrain and pons there was prominent gliosis, microglial activation and vacuolation of the neuropil, which was also seen in the central tegmental tract. In the medulla, the ninth, 10th and 12th cranial nerve nuclei showed severe neuronal depletion and gliosis with pale corresponding nerve tracts. The neuronal loss in the eighth cranial nerve was moderate (Supplementary Fig. 1). The medial lemniscus, spinocerebellar tract and medullary reticular formation were all gliotic. Inferior olivary nuclei, in particular the dorsal and ventral parts, showed severe neuronal loss and gliosis. In the cerebellum, there was no significant cortical atrophy [Supplementary Fig. 2B(i)], but the cerebellar white matter showed widespread vacuolation. In the middle cerebellar peduncle there was a lesion with similar morphology to those seen in the pons and medulla. In the spinal cord, there was moderate neuronal loss in the anterior horns and moderately severe atrophy of spinothalamic tracts, spinocerebellar tracts, gracile fasciculus and nucleus in the medulla, and to a lesser extent cuneate fasciculus and nucleus in the medulla (Supplementary Fig. 1). There was severe fibre loss and macrophage activation in the anterior spinal nerve roots, whilst the poster spinal nerve roots were densely populated by myelinated fibres with little macrophage activation (Fig. 1E–H).

Patient AM4 also had a normal birth at term. He had hearing problems from the age of 8 years and was diagnosed with sensorineural hearing loss at the age of 11. He developed optic atrophy and difficulty walking at the age of 16 years. At age 17 years he presented with dysarthria and subsequently developed swallowing difficulties. EMG showed widespread denervation and nerve conduction studies were consistent with an axonal motor neuropathy. He died at 19 years of respiratory insufficiency.

**Figure 2:**
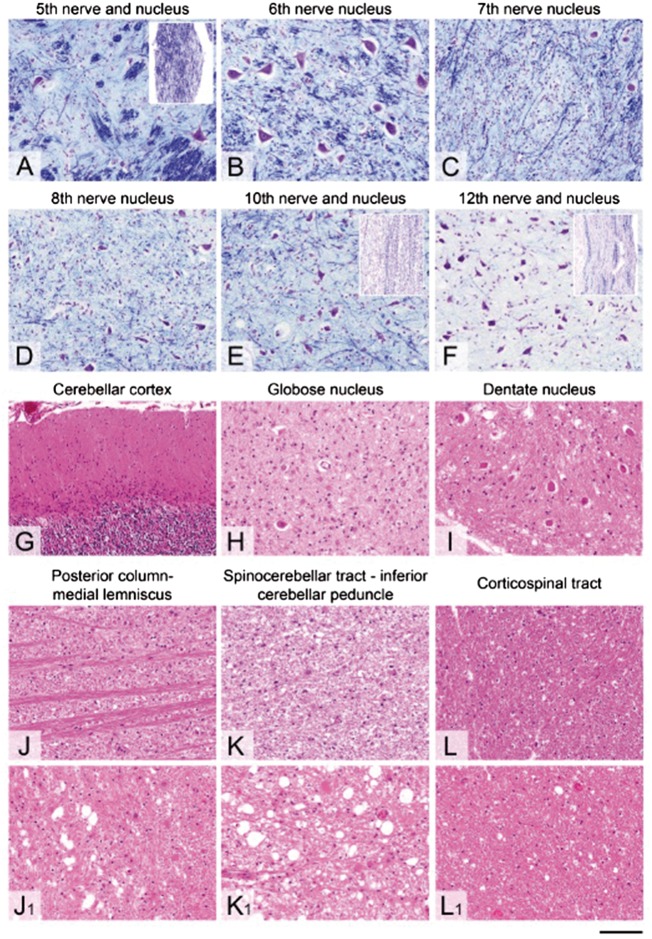
**The spectrum of the atrophy in the cranial nerve nuclei, deep cerebellar nuclei and white matter tracts in Patient AM4.** (**A**) In the fifth cranial nerve nucleus there is a moderately severe neuronal loss and gliosis and accompanying myelinated fibre loss in the nerve tract (*inset*). (**B**) The sixth cranial nerve nucleus shows only very mild neuronal loss and gliosis. (**C**) In the seventh and (**D**) eighth cranial nerve nuclei the neuronal loss and accompanying gliosis is very severe. (**E**) In the nuclei of the 10th and (**F**) 12th cranial nerves the neuronal loss and gliosis is moderately severe, but the nerve tracts (*inset* in **E** for the 10th nerve and *inset* in **F** for the 12th nerve) show marked depletion of myelinated fibres. (**G**) In the cerebellar cortex there is widespread Purkinje cell loss, Bergmann gliosis and gliosis in the molecular cell layer. (**H**) The globose nucleus shows a severe neuronal loss and gliosis, while (**I**) the neurons in the dentate nucleus are better preserved and gliosis is mild. (**J**) The medial lemniscus in the medulla, (**J_1_**) the gracile fasciculus in the posterior column, (**K**) inferior cerebellar peduncle in hindbrain and spinocerebellar tract in the spinal cord (**K_1_**) show severe gliosis and vacuolation of the neuropil on haematoxylin and eosin-stained sections, and microglial activation on immunohistochemistry (not shown). (**L** and **L_1_**) The corticospinal tracts at the level of medulla (**L**) and spinal cord (**L_1_**) in contrast is well populated by myelinated fibres with no apparent gliosis. Scale bar = 110 µm in **A**–**F** and **H**–**L**; 220 µm in **G**. (**A**–**F**) Stained with Luxol® fast blue. (**G**–**I** and **J**, **J_1_**–**L**, **L_1_**) Stained with haematoxylin and eosin.

The cerebral neocortex, hippocampus, amygdala, caudate nucleus putamen, globus pallidus, thalamus and cerebral deep white matter showed no apparent abnormality. Mild gliosis was evident in the dorsal part of the optic tract. In the midbrain, the substantia nigra was densely populated by lightly pigmented neurons in keeping with the patient’s age. The corticospinal tracts were unremarkable in the cerebral peduncles of the midbrain. The third cranial nerve nucleus was not available for the assessment and the fourth cranial nerve nuclei showed a mild degree of neuronal loss and accompanying mild gliosis (Supplementary Fig. 3). In the pons there was prominent neuronal loss in the loci coerulei with free pigment deposits in the neuropil and gliosis (Supplementary Fig. 3). The neuronal density in the pontine basal nuclei and the fibre density in the transverse and corticospinal tracts were unremarkable. The superior cerebellar peduncles showed mild gliosis and vacuolation. The fifth and seventh cranial nerve nuclei showed severe neuronal loss (Supplementary Fig. 3 and Fig. 2A and C). In the sixth cranial nerve nucleus the gliosis was mild and the neuronal density was relatively well-preserved (Fig. 2 and Supplementary Fig. 3). In the medulla (Fig. 2D–F and Supplementary Fig. 3), the nuclei of the eighth cranial nerve showed severe neuronal loss and prominent gliosis and the nuclei of the 10th and 12th cranial nerves and nucleus ambiguus showed moderately severe neuronal loss and gliosis. The nerve tracts of the available fifth, 10th and 12th cranial nerves (Fig. 2A, E and F, insets) showed reduced density of the myelinated fibres. The gracile nucleus showed severe, while the cuneate nucleus showed mild neuronal loss and gliosis (Supplementary Fig. 3). In the cerebellar cortex there was widespread Purkinje cell loss and Bergmann gliosis and some degree of neuronal loss in the granular cell layer [Fig. 2G and Supplementary Fig. 2A(i)].

**Figure 3:**
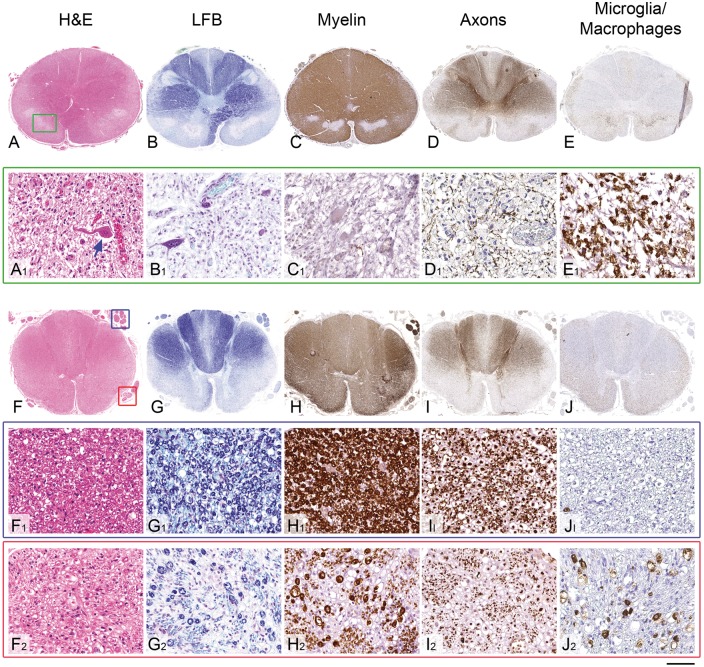
**Pathological features of the symmetrical brain stem and spinal cord lesions and the comparison of the spinal cord nerve root involvement in the Patient AM4.** (**A**–**E**) Low power views of the upper cervical cord and (**A_1_**–**E_1_**) high power views of the lesion indicated with green square box in **A**. (**A** and **A_1_**) The haematoxylin and eosin (H&E) stained section demonstrates bilateral symmetrical sharply demarcated lesions (indicated with a green square box on one side) in the anterior part of the upper cervical cord. High power view of the lesion (**A_1_**) reveals intact neurons (blue arrow) within a dense infiltrate of foamy macrophages and increased numbers of small calibre blood vessels. (**B** and **B_1_**) Staining for myelin with Luxol® fast blue (LFB) special stain and (**C** and **C_1_**) immunostaining with SMI94 antibody highlights the preservation of the neurons and shows complete absence of myelin, whilst the axons (**D** and **D_1_**), demonstrated with SMI31 antibody, are better preserved within the lesion. (**E** and **E_1_**) Dense infiltrates of macrophages within the bilateral lesions are confirmed with CD68 immunohistochemistry. (**F**–**J**) Low power views of transverse section of the thoracic spinal cord. The posterior nerve roots are indicated with blue square box in **F** and on high power views in **F_1_**–**J_1_**. The anterior nerve roots are indicated with red square box in **F** and on high power views in **F_2_**–**J_2_**. (**F_1_**–**I_1_**) The posterior nerve roots are densely populated with myelinated fibres with (**J_1_**) minimal macrophage activity. (**F_2_**–**I_2_**) In the anterior nerve roots there is a moderately severe loss of myelinated fibres and (**J_2_**) prominent macrophage activation. Scale bars = 2.5 mm in **A**–**E** and **F**–**J**; 80 µm in **A_1_**–**E_1_**_,_**F_1_**–**J_1_** and **F_2_**–**J_2_**.

The cerebellar nuclei (fastigii, globosus and emboliformis) showed severe neuronal loss and gliosis bilaterally, while the dentate nuclei were mildly gliotic with less conspicuous neuronal loss (Fig. 2H–I). There was a severe pallor, gliosis and prominent microglial/macrophage activation in the medial lemniscus (Fig. 2J), spinothalamic tracts and inferior cerebellar peduncles (Fig. 2K). The corticospinal tracts in the pyramids (Fig. 2L) were densely populated by myelinated fibres and the inferior olivary nuclei showed only mild patchy gliosis (Supplementary Fig. 3). In the ventral aspect of the upper spinal cord at the junction with the lower medulla there were bilateral symmetric lesions involving the anterior horns, lateral reticular nucleus, supraspinal nucleus, spinothalamic tracts and medial longitudinal fasciculus (Fig. 3A–E). In these lesions there was neovascularization and dense infiltration of macrophages with a near complete loss of myelin, while neurons and axons were relatively preserved (Fig. 3A–E). Below these lesions the cervical cord showed a severe atrophy of the neurons in the anterior horns and to a lesser extent posterior horns (Supplementary Fig. 3). There was a severe, slightly asymmetrical atrophy of the uncrossed anterior corticospinal tracts, whilst the lateral corticospinal tracts were densely populated by myelinated fibres with only mild vacuolation [Fig. 2L(i) and Supplementary Fig. 3]. Severe symmetrical atrophy with prominent pallor, macrophage infiltration and vacuolation of the anterior and posterior spinocerebellar tracts [Fig. 2K(i)], spinothalamic tracts and gracile and to a lesser extent cuneate fasciculi (Supplementary Fig. 3) was also observed. While the posterior nerve roots were densely populated by myelinated fibres with only mild macrophage activation, in the anterior nerve roots there was moderately severe loss of myelinated fibres and prominent infiltration of macrophages (Fig. 3F–J).

Amyloid-β, hyper phosphorylated tau, α-synuclein, TDP-43, p62 or ubiquitin positive inclusions were not observed in Patients AM2 and AM4. In both cases the inflammatory reaction was restricted to microglial activation and macrophage infiltrates with no significant lymphocytic inflammation.

### Generation of a novel *Drosophila* model of BVVL syndrome

Mitochondrial dysfunction has long been documented in neurodegenerative diseases (Palomo and Manfredi, 2015). For example, SOD1 mutations in familial ALS have been shown to lead to abnormalities in mitochondrial morphology, both in biopsies and post-mortem tissues of human patients (Sasaki and Iwata, 2007; Sasaki, 2010) and in cellular and mouse models of the disease (Magrane *et al.*, 2009, 2014; Vinsant *et al.*, 2013; Palomo and Manfredi, 2015). However, whether mitochondrial function is perturbed by BVVL syndrome-linked mutations is unclear. We hypothesized that lower levels of intracellular riboflavin as a result of mutated riboflavin transporters would lead to reduced levels of FMN and FAD, which in turn would lead to impairments at the level of the ETC complex I and complex II. We sought to test this hypothesis *in vivo*.

Previous work has used a knockout of the mouse *SLC52A3* orthologue to model BVVL syndrome (Intoh *et al.*, 2016; Yoshimatsu *et al.*, 2016). However, *SLC52A3* knockout mice die within 48 h of birth, precluding phenotypic analysis at later developmental stages. To circumvent this issue we turned to the fruit fly, *Drosophila*. Comparative BLAST analysis revealed a single *Drosophila* homologue of *SLC52A3/*hRFVT3, the previously uncharacterized gene *cg11576* (E-value: 2.74 × 10^−74^; next closest homologue, E-value: 2.95). As described below, we name the *cg11576* gene *drift* (*Drosophila*riboflavin transporter). The DRIFT amino acid sequence exhibits 36.9% identity and 53.1% similarity with hRFVT3, and the L1 loop and GXXXG motifs characteristic of riboflavin transporters are also conserved (Supplementary Fig. 4) (Russ and Engelman, 2000; Zhang *et al.*, 2010). The L1 loop is a region of the protein shown to recognize riboflavin through both hydrogen bonds and van der Waals interactions (Zhang *et al.*, 2010), while the GXXXG motif is required for dimerization (Russ and Engelman, 2000). DRIFT is also highly homologous to the hRFVT3 paralogues hRFVT1 and hRFVT2 (Supplementary Fig. 4). Furthermore, 10/19 and 11/25 residues in hRFVT2 and hRFVT3, respectively, that are altered by BVVL syndrome-linked mutations are either identical or functionally similar in DRIFT (Supplementary Fig. 4 and 5). Mammalian riboflavin transporters exhibit wide domains of expression, including the nervous system, intestine, testes, and placenta (Yonezawa and Inui, 2013). Similarly, *drift* is transcribed in several adult *Drosophila* tissues, including the head, gut, abdomen and thorax (Supplementary Fig. 6A). This broad expression pattern is in agreement with RNAseq data from the *Drosophila* ModEncode Project (Graveley *et al.*, 2011).

To examine the biochemical and phenotypic consequences of loss of DRIFT, we disrupted *drift* expression using either P-element insertions or transgenic RNA interference (RNAi). Insertion of a MiMIC transposon (*cg11576*^MI04904-GFSTF.2^) within intron 1 of *drift* resulted in viable heterozygote adults but homozygote lethality prior to the L3 larval stage. Furthermore, global knockdown of *drift* using two independent transgenic RNAi lines (7578 and HMC04813) driven via a strong global promoter (*actin*-Gal4) also resulted in either complete or highly penetrant lethality prior to the adult stage. Thus, similarly to *SLC52A3* (Yonezawa and Inui, 2013; Intoh *et al.*, 2016; Yoshimatsu *et al.*, 2016), and consistent with a fundamental requirement for riboflavin transport in metazoans (Biswas *et al.*, 2013), DRIFT is likely to be essential in *Drosophila*.

**Figure 4:**
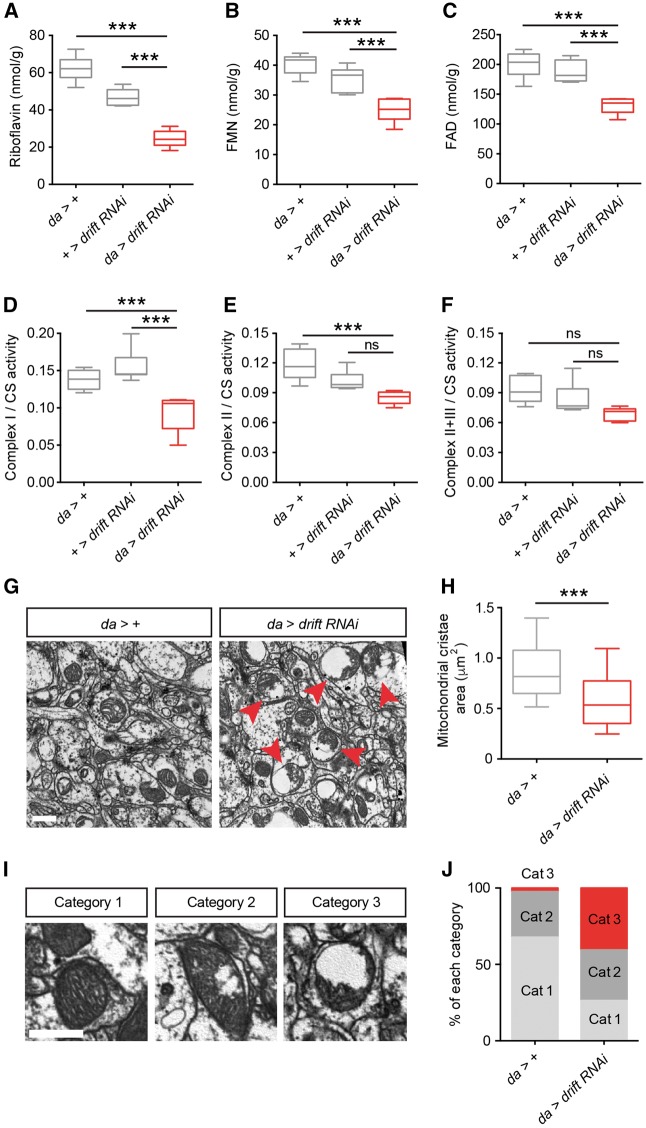
**Knockdown of the *Drosophila* riboflavin transporter homologue *drift* results in reduced ETC activity and abnormal mitochondrial morphology**. (**A**–**C**) Riboflavin (**A**), FMN (**B**) and FAD (**C**) levels in *drift* knockdown flies and controls normalized to total protein levels. (**D–F**) Complex I (**D**), complex II (**E**) and complex II-III (**F**) activity in *drift* knockdown flies and controls. Complex activities are normalized to citrate synthase (CS) activity. Data were generated from a minimum of three independent experiments; *n* = 10 for each genotype. Individual measurements were performed in duplicates. (**G**) Representative electron micrographs showing ultrastructure of neuronal mitochondrial in *drift* knockdown and control adult fly brains. Red arrows indicate severely damaged mitochondria. Scale bar = 1 μm. (**H**) Neuronal *drift* knockdown mitochondria display significant reductions in the area of the mitochondrial cristae. *da > +* control: *n* = 295 mitochondria; *drift* knockdown: *n* = 516. (**I**–**J**) Morphological analysis also reveals a larger proportion of abnormal mitochondria in the *drift* knockdown fly brains compared to controls. Representative images of respective categories are shown in **I**. Scale bar = 0.5 μm. Proportion of mitochondria in each category is shown in **J**. *da > +* control: *n* = 295 mitochondria; *drift* knockdown: *n* = 516. For **A**–**F** and **H**, data are presented as box plots illustrating 80% of the data distribution. Tenth, 25th, median, 75th and 90th percentiles are shown for these and all subsequent box plots. ***P < 0.0005, ns = *P* > 0.05, one-way ANOVA with Dunnett’s *post hoc* test (**A**–**F**) or Mann-Whitney U-test (**H**).

Using a distinct global driver (*daughterless*-Gal4; *da*-Gal4) in concert with the 7578 RNAi line, we achieved robust *drift* knockdown as determined by semi-quantitative RT-PCR (Supplementary Fig. 6C). However, this knockdown did not result in early lethality (suggesting weaker RNAi expression by *da*-Gal4 relative to *actin*-Gal4), facilitating analysis of *drift* knockdown flies at later developmental stages. Using whole-body tissue from *drift* knockdown adults and associated control lines (heterozygotes for *da*-Gal4 or the *drift* RNAi transgene), we found that *drift* knockdown resulted in a substantial reduction of *in vivo* riboflavin levels as well as the riboflavin metabolites FMN and FAD (Fig. 4A–C). These results, combined with the high homology of DRIFT to hRFVT1–3, strongly suggest that DRIFT is a *bona fide* riboflavin transporter.

We next asked whether *drift* knockdown resulted in reduced mitochondrial activity. Indeed, ETC complex I activity was significantly reduced by *drift* knockdown (Fig. 4D), while ETC complex II and II-III activity exhibit a trend towards lower levels, albeit non-significant (Fig. 4E and F). Thus, ETC complex I activity appears particularly sensitive to DRIFT expression.

We also tested whether similar reductions in riboflavin metabolism and ETC activity could be observed in BVVL syndrome patient tissue. Using fibroblasts derived from BVVL syndrome patients with riboflavin transporter mutations and healthy age-matched controls (Supplementary material), we found a significant reduction in the intracellular levels of FMN and FAD in patient fibroblasts when grown in low extracellular riboflavin conditions (Supplementary Fig. 7A and B). Levels of intracellular riboflavin in patient fibroblasts frequently fell below the threshold of detection under these conditions, but not in control fibroblasts (data not shown), consistent with defective riboflavin transport. Furthermore, we observed a significant reduction in ETC complex I and complex II activity in patient fibroblasts compared to controls (Supplementary Fig. 7C and D). Taken together, the above *in vivo* and *in vitro* data suggest a conserved role for riboflavin transporters in regulating ETC activity, particularly ETC complex I.

### *drift* knockdown lowers mitochondrial membrane potential and affects respiratory chain activity

Given the reduced ETC complex I activity in *drift* knockdown flies, we investigated whether loss of *drift* affected mitochondrial membrane potential (ΔΨ_m_), an indicator of mitochondrial health and function. Mitochondrial membrane potential was assessed in the brains of 1-day-old flies using tetramethylrhodamine, methyl ester (TMRM) fluorescence and imaging with confocal microscopy. This revealed a significant reduction in mitochondrial membrane potential in *drift* knockdown brains compared to control (Supplementary Fig. 8A).

We next isolated mitochondria from *drift* knockdown and control flies and assessed respiratory chain activity by measuring oxygen consumption using a Clark-type oxygen electrode. The steps in respiration were compared using substrates and inhibitors specific to individual respiratory complexes. We found that *drift* knockdown leads to significantly increased oxygen consumption in state 2 (ADP independent) respiration, when using substrates for complex I (5 mM pyruvate and 5 mM malate) but not substrates for complex II (5 mM succinate in the presence of 5 µM rotenone; Supplementary Fig. 8B). The respiratory control ratio (RCR) is the ratio of state 3 respiration (ADP stimulated) to state 4 respiration (no ADP present), and is considered an indication of the degree of coupling of mitochondrial respiratory chain activity to oxidative phosphorylation (Chance and Williams, 1955). RCR was significantly lower in *drift* knockdown mitochondria compared to control flies (Supplementary Fig. 8C). Furthermore, the efficiency of oxidative phosphorylation (measured as ratio ADP/O) was also significantly lower in *drift* knockdown mitochondria (Supplementary Fig. 8D). Collectively, these results suggest that loss of *drift* activity reduces mitochondrial membrane potential as a result of mitochondrial uncoupling.

### DRIFT is required for normal mitochondrial morphology

Given the alterations in mitochondrial function resulting from reduced DRIFT levels, we also assessed whether mitochondrial morphology was regulated by DRIFT. To do so, we used electron microscopy to enable ultrastructural examination of mitochondria within the neuropil regions (axons, dendrites and synaptic domains) of the adult nervous system in *drift* knockdown flies and an associated control. Strikingly, we frequently observed grossly abnormal mitochondria containing swollen vacuoles in *drift* knockdown neurons, coupled with fragmented mitochondrial cristae and a concomitant reduction in the area of mitochondrial cristae (Fig. 4G and H). Grouping mitochondria from control and *drift* knockdown neurons in three categories (exhibiting no, minor, or major mitochondrial swelling and cristae fragmentation) (Fig. 4I), we found that morphologically normal mitochondria were depleted in *drift* knockdown neurons, whereas the proportion of mitochondria exhibiting gross structural defects was greatly increased (Fig. 4J). Thus, we have identified a profound neuronal endophenotype associated with reduced riboflavin transporter expression: vacuolar mitochondria with fragmented cristae.

### *drift* knockdown results in reduced locomotion and lifespan in *Drosophila*

**Figure 5:**
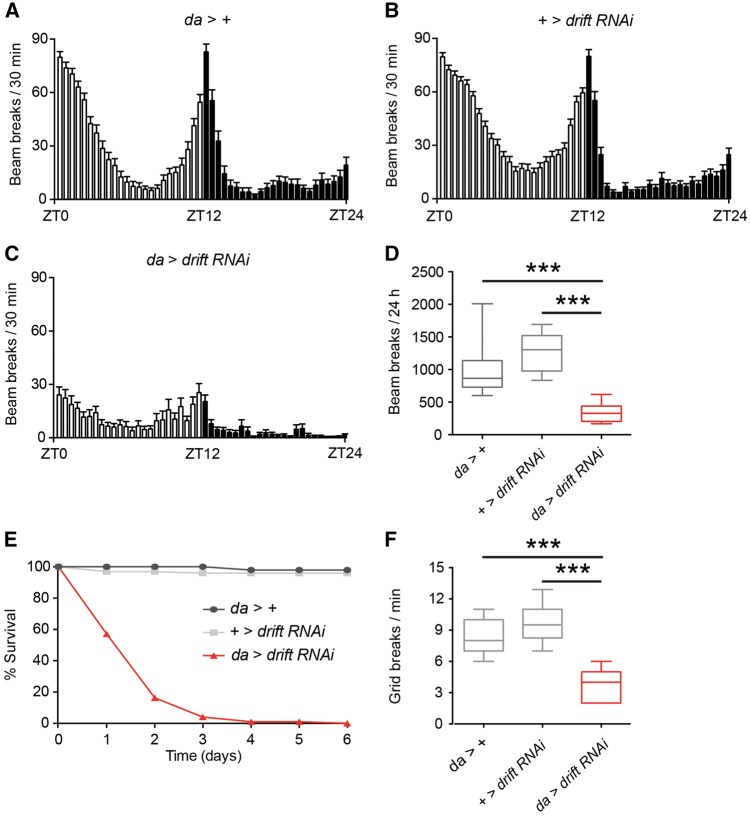
***drift* knockdown reduces locomotor activity and lifespan in adult *Drosophila*.** (**A**–**C**) Locomotor activity over 24 h of driver alone (**A**) and *drift* RNAi alone (**B**) controls, and *drift* knockdown flies (**C**). Data were derived from adult females. Mean values for each time point are presented; error bars indicate standard error of the mean (SEM). ZT = zeitgeber time. Light bars: lights on, darks bars: lights off. (**D**) Total activity over 24 h for each genotype. Data were generated in at least three independent experiments; *n* = 30 for each control and *n* = 16 for the *drift* knockdown flies. (**E**) Percentage survival of *drift* knockdown flies and controls on normal food; *n* = 98 for each control and *n* = 99 for the *drift* knockdown flies. (**F**) The number of grid breaks per min of wandering third instar larvae; *n* = 30 for each genotype. ***P < 0.0005, Kruskal-Wallis test with Dunn’s *post hoc* test.

The viability resulting from *drift* knockdown via *da*-Gal4 allowed us to assess whether riboflavin transporter knockdown impacts post-embryonic organismal phenotypes in a manner consistent with BVVL syndrome pathology (Foley *et al.*, 2014; Manole *et al.*, 2014). To test for locomotor defects, we used the *Drosophila* activity monitor (DAM) system to perform automated recordings of adult locomotion, measured as the number of infra-red beam breaks across a 24 h day/night cycle. Under 12 h light: 12 h dark conditions, control 1–2 day old adult female *Drosophila* exhibited peaks of activity at dawn and dusk, and relative quiescence during the afternoon and night (Fig. 5A and B). In contrast, peak activity and total beam breaks in *drift* knockdown adults were substantially reduced (Fig. 5C and D). Furthermore, *drift* knockdown resulted in greatly reduced lifespan, with 99% mortality within 4 days post-eclosion (Fig. 5E). As BVVL syndrome is an early-onset disorder, we also tested for locomotor defects at earlier stages of the *Drosophila* lifecycle. Indeed, we found that *drift* knockdown third instar larvae also exhibit significantly reduced locomotion, as measured by the number of grid crosses per minute on an agar plate (Fig. 5F). These *Drosophila* phenotypes mimic motor dysfunction and early mortality observed in patients with BVVL syndrome, suggesting a conserved link between riboflavin transporter dysfunction, locomotor strength and lifespan.

Recently, reductions in axonal length and transcriptional signatures of altered cytoskeletal composition were identified in BVVL syndrome iPSC-derived motor neurons (Rizzo *et al.*, 2017). To test for altered axonal development and signatures of cytoskeletal dysfunction *in vivo*, we examined the neuromuscular junction of *drift* knockdown third instar larvae. Interestingly, despite the clear reduction in locomotion in *drift* knockdown larvae (Fig. 5F), motor neuron development appeared unperturbed. Motor neuron axons correctly innervated the muscle surface and there was no reduction in the number of synaptic boutons per motor neuron following *drift* knockdown (Supplementary Fig. 9A and B). Mutations in cytoskeletal components such as Ankyrin, Spectrin, HTS/Adducin and Futsch (a microtubule-binding protein) cause well-defined alterations in the morphology of larval motor neurons. These include synaptic retraction (disassembly of active zones and degradation of inter-bouton axonal regions, leaving small isolated boutons) (Pielage *et al.*, 2005, 2008, 2011), aberrant filopodia-like protrusions (Pielage *et al.*, 2011), or a reduction in synaptic growth (Roos *et al.*, 2000). As we did not observe similar phenotypes at the neuromuscular junction of *drift* knockdown larvae, we infer that cytoskeletal organisation is grossly normal in this background, and therefore that locomotor defects can initially occur in the absence of altered synaptic development and/or neurodegeneration following *drift* knockdown.

### An esterified riboflavin derivative rescues *drift* knockdown phenotypes

**Figure 6:**
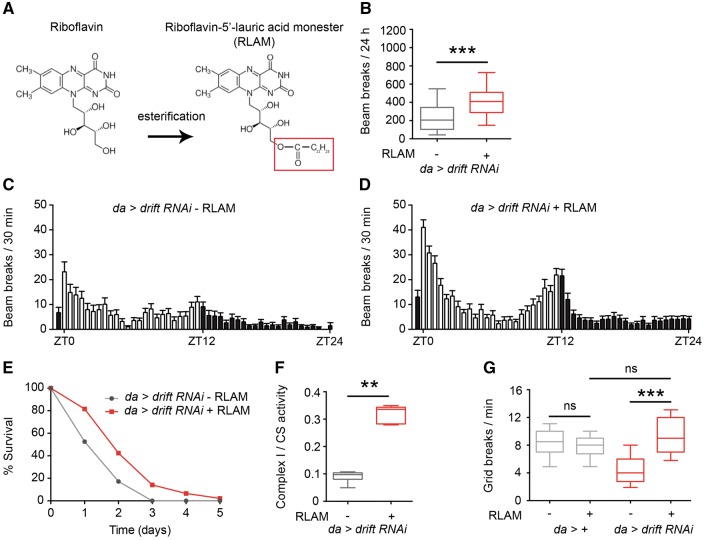
**A riboflavin ester partially rescues the *drift* knockdown phenotypes.** (**A**) Chemical structure of riboflavin and its ester (RLAM). (**B**) Total locomotor activity over 24 h in *drift* knockdown grown on normal and RLAM-supplemented food. Data were generated in at least three independent experiments; *n* = 37 and *n* = 52 *drift* knockdown given normal and RLAM-supplemented food, respectively. (**C** and **D**) Mean locomotor activity over 24 h of *drift* knockdown flies grown on normal food (**C**) and RLAM-supplemented food (**D**). Values are presented as a mean ± SEM. ZT = zeitgeber time. Light bars: lights on, darks bars: lights off. (**E**) Percentage survival of *drift* knockdown flies grown on normal and RLAM-supplemented food; *n* = 99 and *n* = 92 *drift* knockdown given normal and RLAM-supplemented food, respectively. (**F**) Complex I activity in *drift* knockdown flies grown on normal and RLAM-supplemented food. Complex activity is expressed as a ratio to citrate synthase (CS) activity. Data were generated in at least three independent experiments; *n* = 10 for each genotype. (**G**) The number of grid breaks per min of wandering third instar larvae with and without RLAM supplementation; *n* = 20 for each genotype. **P < 0.005, ***P < 0.0005, ns = *P* > 0.05, Mann-Whitney U-test (**B** and **F**) or Kruskal-Wallis test with Dunn’s *post hoc* test (**G**).

Since BVVL syndrome pathology can be partially ameliorated by riboflavin treatment, we asked whether locomotor defects in *drift* knockdown flies could be rescued by supplementing *Drosophila* culture medium with riboflavin (0.1 mg/ml). However, we found no enhancement of locomotor activity following riboflavin supplementation (Supplementary Fig. 10A). Riboflavin is a water-soluble vitamin that is easily excreted, leading to low bioavailability and short half-life. Furthermore, since riboflavin transporter expression is very low in *drift* knockdown flies (Supplementary Fig. 6C), riboflavin in the *Drosophila* haemolymph may fail to be transported into relevant cell types. We sought to circumvent these issues using an esterified derivative of riboflavin (riboflavin-5’-lauric acid monoester; RLAM; 0.1 mg/ml) that could act as a pro-drug, likely diffuse into the intracellular space independently of riboflavin transporter function and be cleaved by esterases to release active riboflavin (Fig. 6A). Strikingly, food supplementation with RLAM resulted in heightened locomotion (Fig. 6B–D) and an extension of lifespan (Fig. 6E) in *drift* knockdown flies. This rescue correlated with substantially increased complex I activity (∼3-fold) following RLAM supplementation (Fig. 6F). In contrast to *drift* knockdown flies, RLAM did not induce a significant fold-change in locomotor activity in control adults, suggesting that RLAM is non-toxic at the concentration and time scale of supplementation tested (Supplementary Fig. 10B).

The rescue of adult *drift* knockdown by RLAM is clearly partial. For example, activity in *drift* knockdown flies fed RLAM was still significantly lower than control flies fed RLAM (beams breaks/24 h: *da* > + controls = 1457 ± 58, *n* = 47; *da* > *drift* RNAi = 415 ± 27, *n* = 53; *P* < 0.0001, Mann-Whitney U-test). We speculated that this partial rescue might be due to the fact that during pupariation, a period of substantial morphological growth and remodelling in the lifecycle of holometabolous insects, pupae will not be exposed to RLAM. Reduced intracellular riboflavin concentrations during pupariation may in turn impact critical aspects of development, resulting in adult flies that can only be partially rescued by RLAM supplementation. If true, we would expect to observe a more robust rescue with RLAM during earlier phases of development, when larval *Drosophila* are continually exposed to RLAM in the food. Indeed, in *drift* knockdown L3 larvae fed RLAM, we observed a rescue of larval locomotor activity to wild-type levels (Fig. 6G). Similarly to adult flies, riboflavin supplementation had no effect on the activity of *drift* knockdown L3 larvae (Supplementary Fig. 10C). Thus, we hypothesize that RLAM may represent a more efficient treatment method for BVVL syndrome patients, since—in contrast to riboflavin—cellular uptake of RLAM may still robustly occur in the absence of functional riboflavin transporters.

## Discussion

We expanded the spectrum of genetic defects in riboflavin transporters identifying *SLC52A2* and *SLC52A3* mutations in 15% of cases. It was previously noted that the distinct phenotype of upper limb and axial weakness, hearing loss and optic atrophy could be attributed only to patients harbouring *SLC52A2* mutations (Foley *et al.*, 2014). However, in our cohort of 20 patients, mutations in both *SLC52A2* and *SLC52A3* resulted in similar phenotypes. *SLC52A3* mutations were more frequent than mutations in *SLC52A2* in our cohort. This is in agreement with previous reports where BVVL syndrome-linked mutations were predominantly found in *SLC52A3* (Bosch *et al.*, 2011; Manole and Houlden, 2015). The high proportion of patients negative for mutations in the known riboflavin transporters indicates that novel genetic causes are yet to be found.

The neuropathology of BVVL syndrome has not been fully characterized (Sathasivam, 2008; Malafronte *et al.*, 2013). In keeping with the spectrum of clinical findings, severe depletion of neurons in multiple cranial nerve nuclei, anterior horns of the spinal cord, cerebellar nuclei, Purkinje cells in the cerebellum, and degeneration of optic pathway, solitary tract, and spinocerebellar and pyramidal tracts was seen along with an axonal neuropathy in the sural nerve (Foley *et al.*, 2014). Here we provide detailed neuropathological assessment of the brains and spinal cords in two patients with genetically confirmed mutations in the *SLC52A3* gene who fell at the two extremes of age of presentation of BVVL syndrome. There was variably and severe neuronal loss and gliosis in the brain stem cranial nerve nuclei and anterior horns of the spinal cord with accompanying nerve root atrophy, which reflects the spectrum of clinical symptomatology. While no correlation between the mutation type and severity of clinical phenotypes was evident in the case series presented here, it is possible that different mutations directly influence the length of the disease and degree of atrophy of specific brain structures. For example, the rapid deterioration and demise of Patient AM2 due to formation of truncated protein originating from nonsense mutations on both alleles may explain less prominent atrophy of cerebellar Purkinje cells when compared with Patient AM4, the atrophy of which may require longer duration of the disease (Supplementary Fig. 2). In both patients there were symmetrical lesions in the brain stem characterized by prominent neovascularization, dense infiltration of macrophages, loss of myelin and relative preservation of neurons and axons. Although the anatomical distribution of the symmetrical lesions differed in both cases (Supplementary Fig. 1 and 3), the morphology of the lesions was identical in both cases and was similar to the pathology seen in mitochondrial encephalopathies (Tanji *et al.*, 2001; Filosto *et al.*, 2007). To the best of our knowledge such lesions have not been documented in any of the previous published cases of BVVL clinical syndrome and link with the mitochondrial abnormalities in *Drosophila* and patient fibroblasts.

Recent work has sought to model the effects of riboflavin transporter mutations in iPSC-derived motor neurons from patients with BVVL syndrome (Rizzo *et al.*, 2017) or *SLC52A3* knockout mice (Intoh *et al.*, 2016; Yoshimatsu *et al.*, 2016). Motor neurons can still be successfully differentiated from embryonic stem cells derived from mice lacking *SLC52A3* (Intoh *et al.*, 2016), suggesting that RFVT3 (the *SLC52A3* gene product) is not required for motor neuron development in culture. In contrast, Rizzo *et al.*, (2017) recently identified reduced axonal growth and transcriptional alterations in cytoskeletal factors, including neurofilament genes, in iPSCS-derived motor neurons from *SLC52A3-*BVVL syndrome patient cells. Our results are inconsistent with the premise that cytoskeletal defects occur in motor neurons of *Drosophila* larvae with reduced DRIFT, and suggest that DRIFT is not required for development of larval motor neurons, similar to findings for the *SLC52A3* knockout mouse (Intoh *et al.*, 2016). We note that the *Drosophila* genome lacks neurofilament genes, preventing us from assessing the potential relevance of this pathway to neuronal dysfunction in BVVL syndrome model *Drosophila*. However, the fact that locomotor defects and reduced lifespan still occur in *drift* knockdown flies in the absence of neurofilaments suggest that additional pathways are likely to contribute to neuronal dysfunction following loss of riboflavin transporter in flies, and potentially mammals. Based on our findings in both patient fibroblasts and *Drosophila*, we suggest that mitochondrial dysfunction may represent one such pathway that impacts neuronal signalling and viability in patients with BVVL syndrome.

Although fibroblasts are non-neural cells and not particularly vulnerable as a tissue, metabolic and mitochondrial abnormalities are commonly studied in this cell type (Distelmaier *et al.*, 2009). We identified clear deficiencies in the activities of ETC complex I and complex II in patient-derived fibroblasts relative to fibroblasts from healthy controls. Interestingly, previous results have only shown evidence of marginally decreased ETC complex I activity in muscle cells from some *SLC52A2* patients sensitive to reduced riboflavin uptake (Foley *et al.*, 2014). Mitochondrial activity in different tissues and cell types may thus be differentially sensitive to reduced riboflavin uptake. Biochemical analysis of *Drosophila drift* knockdown tissue is consistent with data derived from patient fibroblasts, showing diminished levels of riboflavin, FMN and FAD, and reduced complex I activity when expression of the single *Drosophila* riboflavin transporter is perturbed. This in turn led to reduced mitochondrial membrane potential and abnormal mitochondrial respiratory chain activity.

Strikingly, we also observed profound abnormalities in mitochondrial ultrastructure in *Drosophila* neurons following *drift* knockdown. In contrast to the *Drosophila* genome, which encodes a single riboflavin transporter, the human genome contains three riboflavin transporter paralogs that exhibit partially overlapping expression domains, including within the brain (Yonezawa and Inui, 2013; www.human.brain-map.org). Given the potential for functional redundancy between riboflavin transporters co-expressed in the same neuron, we hypothesize that similar alterations in mitochondrial function and morphology in patients with BVVL syndrome are most likely to occur in neurons in which a single mutated riboflavin transporter is expressed. It will be intriguing to document neuronal subtypes with such unique riboflavin transporter signatures and to assess whether these are preferentially sensitive to corresponding riboflavin transporter mutations.

At the whole-organismal level, there was impairment in locomotion at both the larval and adult stages of *drift* knockdown flies, reminiscent of the limb weakness and movement impairment of the BVVL syndrome patients. Moreover, *drift* knockdown flies had severely reduced life span, similar to untreated patients (Bosch *et al.*, 2011). It is interesting to note that these lifespan and locomotor defects are rescued by ingestion of an esterified derivative of riboflavin, since this provides further evidence that the phenotypic signatures of *drift* knockdown flies are linked to riboflavin deficiency and consequent downstream metabolic defects. RLAM fulfils the criteria for being a treatment targeting energy metabolism, a fundamental mitochondrial process, in addition to circumventing the disease-related protein, features generally believed to be of great promise (Lin and Beal, 2006; Xuan *et al.*, 2013; Foley *et al.*, 2014).

It was previously thought that defective mitochondria occurs secondary to primary disease mechanism, but current research suggests that mitochondrial dysfunction may play a role in both onset and development of certain neurodegenerative diseases such as Alzheimer’s disease, Parkinson’s disease, Huntington’s disease and ALS, where decreases in one or more mitochondrial complexes and corresponding oxidative stress have been reported in cell or animal models (Menzies *et al.*, 2002; Hoglinger *et al.*, 2003; Fukui and Moraes, 2007; McInnes, 2013; Yamada *et al.*, 2014; Golpich *et al.*, 2017). Our results from *in vitro* and *in vivo* models of a childhood neuropathy are in line with these findings, highlight a potential contribution of mitochondrial dysfunction to BVVL syndrome pathology, and suggest future therapeutic strategies based on esterified riboflavin.

## Supplementary Material

Supplementary Figures

## Abbreviations

ALS: amyotrophic lateral sclerosis
BVVL: Brown-Vialetto-Van Laere
ETC: electron transport chain
FAD: flavin adenine dinucleotide
FMN: flavin mononucleotide
RLAM: riboflavin-5'-lauric acid monoester
