# Supplementary material for: Clinical, pathological and functional characterization of riboflavin-responsive neuropathy
Source: Brain. 2017 Sep 26;140(11):2820–37. doi: 10.1093/brain/awx231 (PMC5808726; doi:10.1093/brain/awx231)
Supplement: Supplementary Figures [file manole_et_al_si_300817this_3008_awx231.pdf]

Clinical, pathological and functional characterization of riboflavin-responsive neuropathy

Andreea Manole,[1,2] Zane Jaunmuktane,[3] Iain Hargreaves,[4] Marthe H. R. Ludtmann,[1] Vincenzo Salpietro,[1,2] Oscar D. Bello,[5] Simon Pope,[4] Amelie Pandraud,[1,2] Alejandro Horga,[2] Renata S. Scalco,[2] Abi Li,[6] Balasubramaniem Ashokkumar,[1,7] Charles M. Lourenço,[8] Simon Heales,[9] Rita Horvath,[10] Patrick F. Chinnery,[11,12] Camilo Toro,[13] Andrew B. Singleton,[14] Thomas S. Jacques,[15] Andrey Y. Abramov,[1] Francesco Muntoni,[16] Michael G. Hanna,[1,2] Mary M. Reilly,[1,2] Tamas Revesz,[1,6] Dimitri M. Kullmann,[5] James E.C. Jepson[5] and Henry Houlden[1,2]

Supplemental Methods, Supplemental Figures S1-S10 and Supplementary References

Supplementary methods

Generation of human fibroblast cultures and cell culture

Skin fibroblasts of BVVL syndrome patients with *SLC52A2* mutations were generated at the Medical Research Council (MRC) Centre for Neuromuscular Diseases Biobank, Dubowitz Centre, UCL Institute of Child Health (ICH) by Dr Diana Johnson, or sent in culture by collaborators. Three age-matched controls were obtained from the ICH Biobank: Control 1 (C1) (age at biopsy: 14 years; female); Control 2 (C2) (age at biopsy: nine years; male); Control 3 (C3) (age at biopsy: five years; male). Five BVVL syndrome patients' fibroblast lines were available for this study. Patients carried the following mutations: E1:p.[Gly306Arg];[Gly306Arg](age at biopsy: 11 years; female); E2:p.[Trp31Ser];[Leu312Pro] (age at biopsy: 7 years; female);E3:p.[Gln234Stop];[Ala420Thr] (age at biopsy: 6 years; female); E4:p.[Gly306Arg];[Leu339Pro] (age at biopsy: 8 years; female); I1: p.[Tyr305Cys]; [Gly306Arg] (age at biopsy: 4 years; male). All cells were maintained at 37°C and 5% CO_2 under humidified conditions and cultured in high glucose Dulbecco's modified Eagle medium (Invitrogen) supplemented with 10% foetal bovine serum (Biowest). All fibroblast lines were grown for four days in modified DMEM containing physiological concentrations (12.6 nM) of riboflavin, followed by four days in modified DMEM, which was either riboflavin-supplemented (300.6 nM) or contained a low riboflavin concentration (3.1 nM).

Determination of Flavin status in fibroblasts

Riboflavin, FMN and FAD content were measured in neutralized perchloric extracts by means of High Performance Liquid Chromatography (HPLC), as previously described (Giancaspero et al., 2009). Quantitative determination of riboflavin, FMN, and FAD was carried out using the Whole Blood Chromsystems vitamins B_1/B_2 kit (Chromsystems, Germany) as per the manufacturer's protocol. The Bio-Rad DC protein assay (Bio-Rad Laboratories, USA) was used to normalise for protein concentration.

Assessment of ETC complex I, II, and citrate synthase activities in fibroblasts

All enzyme activities were determined at 30°C. Prior to analysis all samples were subjected to three cycles of freeze/thawing to lyse membranes. Enzymatic activities were determined using an Uvikon 940 spectrophotometer (Kontron Instruments Ltd, Watford, UK). Complex I activity was measured according to the method of (Ragan, 1987), which involved monitoring the oxidation of NADH at 340 nm. Complex II assay was measured according to the method of (Birch-Machin et al., 1994), which monitored the succinate-dependent 2-thenoyltriflouroacetone sensitive reduction of 2,6-dichlorophenolindophenol at 600 nm. The activity of citrate synthase was measured by the formation of the anion of thionitrobenzoate from 5,5′-dithiobis(2-nitrobenzoate) and CoA at 412

nm (Shephard, 1969). This provided an estimate of mitochondrial content and was therefore used to normalise complex I and II activities for mitochondrial enrichment (Hargreaves et al., 1999).

Semi-quantitative RT-PCR for whole flies

Total RNA was extracted from flies using TRIzol, as per manufacturer's instructions. The concentration and purity of RNA was determined spectrophotometrically. 1 µg of RNA was reverse transcribed to first strand cDNA by using random primers and Moloney murine leukemia virus reverse transcriptase (Promega, Madison, WI, USA). Primers used for *cg11576* (*drift*) were designed so that they span exon-exon boundaries, Forward– 5' CCAGATGCTCCTCTCTCGA 3' Reverse– 5' AGTACACAGTCGCCACTCTC 3'. Primers used for *Drosophila rp49* were: Forward– 5' CCCAACCTGCTTCAAGATGAC 3', Reverse – 5' CCGTTGGGGTTGGTGAGG 3'. GoTaq® Green Master Mix was used and PCR reactions were performed with the following protocol: 95°C-2 min, (95°C-30 s, 60°C-30 s, 73°C-1 min) for 35 cycles, 73°C-5 min, and 4°C-hold. Two exponential curves representing the product formation was made for both primer pairs and cycles 26 and 29 were chosen for *rp49* gene and *drift* respectively so that amplification rates were in the linear range for semi-quantitative comparisons.

Immuno-histochemistry of the *Drosophila* larval neuromuscular junction

When analysing development of the larval neuromuscular junction (NMJ), synapses innervating muscle 6/7, segment 3, were assessed. Confocal z-stacks were taken on a Zeiss confocal LSM710 with a Plan-Apochromat 20x 0.8 NA objective. Late-stage L3 larvae were dissected in low Ca^{2+} (0.2 mM) HL3.1 solution (70 mM NaCl, 5 mM KCl, 20 mM $MgCl_2$, 10 mM $NaHCO_3$, 5 mM trehalose, 115 mM sucrose, 5 mM HEPES, pH 7.2). Dissected NMJ preparations were fixed with Bouin's solution for 10-20 min at room temperature. An alexa-Fluor 488-conjugated goat anti-HRP antibody (1:200; Jackson ImmunoResearch) was used to label the presynaptic neuronal membrane. The post-synaptic subsynaptic reticulem was labelled with mouse anti-Discs Large (4D3, 1:200; Developmental Studies Hybridoma Bank). Both type 1b and type 1s boutons were included in the total bouton count.

Measurement of mitochondrial membrane potential

For measurements of mitochondrial membrane potential ($\Delta\Psi_m$), dissected fly brains were loaded with 25 nM tetramethylrhodamine methylester (TMRM) for 30 min at room temperature and the dye was present for the duration of the experiment. TMRM is used in the redistribution mode to assess $\Delta\Psi_m$, and therefore a reduction in TMRM fluorescence represents depolarization.

Isolation of *Drosophila* mitochondria and measurement of mitochondrial respiration

Fly mitochondria were isolated by differential centrifugation according to (Kinghorn et al., 2015). Briefly, adult flies were chilled on ice and gently pressed using a pre-chilled homogenizer. For each

biological replicate, ~ 150 flies were used. Flies were then washed in isolation buffer (320 mM sucrose, 10 mM Tris/HCl, 10 mM EDTA, pH 7.3) (4°C) (Ferguson et al., 2005).The collected pulp was centrifuged for 10 min at 4°C at 1500 rpm to remove debris. The supernatant was then centrifuged for 10 min at 4°C and 10 000 rpm to collect the mitochondria. The mitochondrial pellet was suspended in respiration buffer (120 mM KCl, 5 mM KH_2PO_4, 3 mM Hepes, 1 mM EGTA, 1mM $MgCl_2$, pH 7.2) and stored on ice. A BCA assay (Sigma) was used to determine the protein concentration of the mitochondrial preparations. The isolated mitochondria were kept on ice and used immediately for experiments. Oxygen consumption was measured using a Clark-type oxygen electrode (Hansatech) thermostatically maintained at 28°C. Isolated mitochondrial were added to respiration buffer and the temperature was maintained at 28°C. Pyruvate (10 mM) and malate (10 mM) were added to measure complex I-linked respiration, and succinate (10 mM) with rotenone (1 µM) were added to measure complex II-linked respiration. 50 nmoles ADP were added to assess ADP:O. All data were obtained using an OxyTherm system (HansaTech) with Chart recording software.

Supplementary Figures

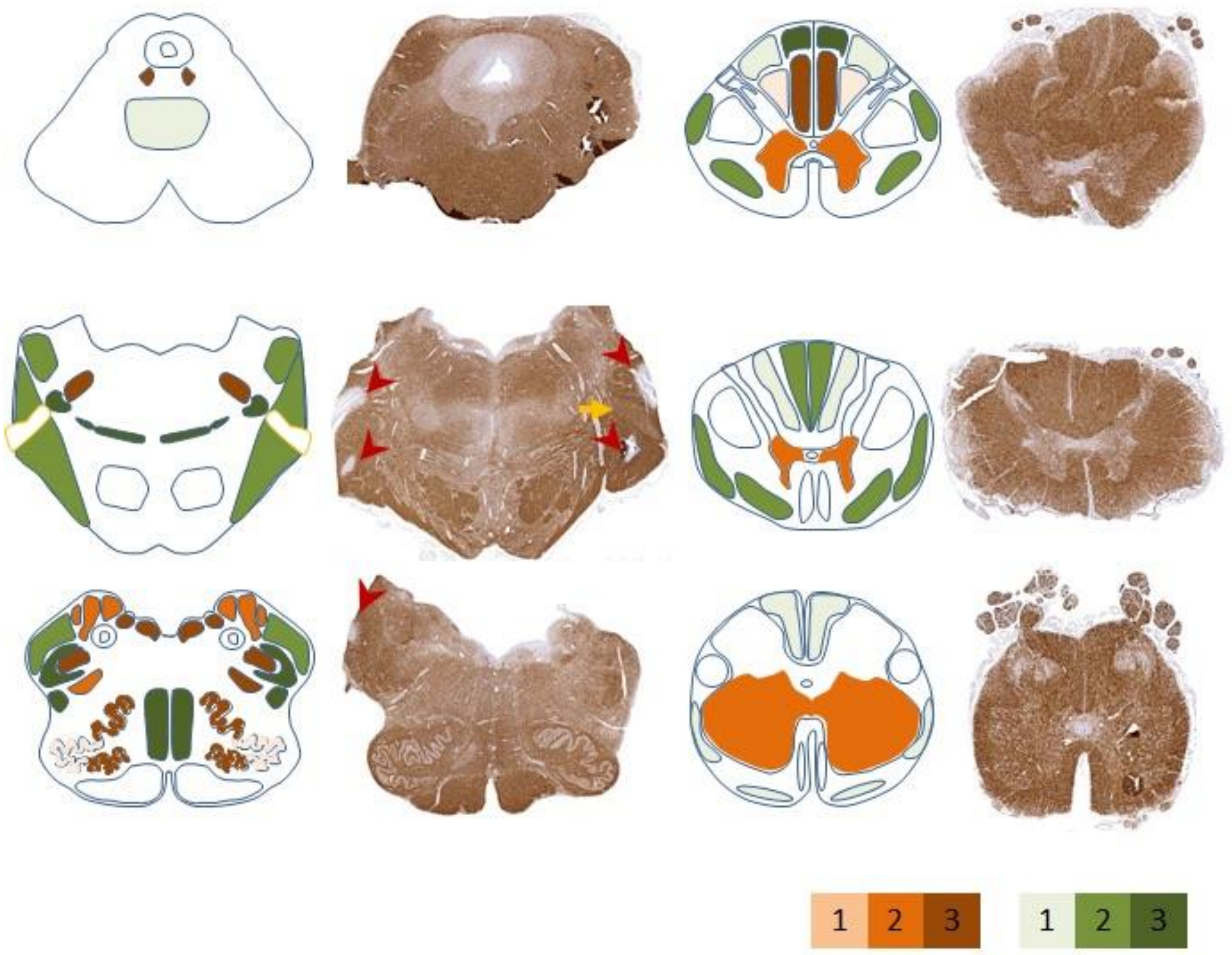

Figure S1. Schematic representation of the anatomical distribution and severity of the brain stem and spinal cord pathology in the patient AM2.

In the schematic figures the severity of grey matter pathology is indicated in orange with the lightest shade corresponding to mild neuronal atrophy and mild gliosis and the darkest shade corresponding to severe atrophy and gliosis. The severity of white matter tract pathology is indicated in green with the lightest shade corresponding to mild pathology and the darkest shade corresponding to a severe myelinated fibre loss. The corresponding transverse brain stem and spinal cord sections are immuno-stained for myelin with SMI94 antibody. The red arrowheads in the pons indicate the bilateral symmetrical lesions surrounding both 5th cranial nerves, indicated with a yellow arrow on one side. The lesion in the medulla is indicated with a red arrowhead.

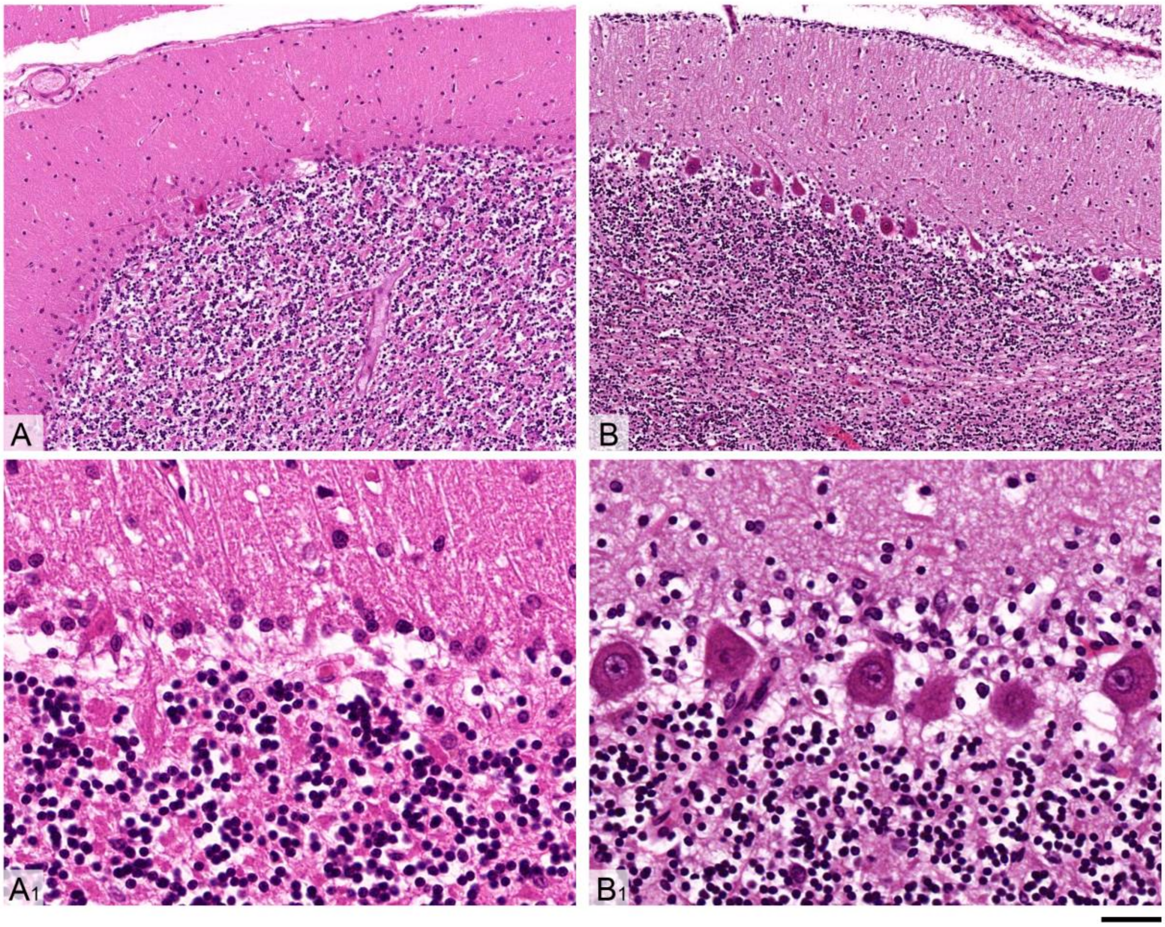

Figure S2. Comparison of the cerebellar atrophy between patient AM4 and AM2

(A and A_1) correspond to patient AM4 and (B and B_1) correspond to patient AM2. (A and A_1) In the cerebellar cortex from the patient AM4 there is severe Purkinje cell loss with widespread Bergmann gliosis. (B and B_1) In the patient AM2 the Purkinje cells are well preserved. Note the presence of external granular cell layer (B_1) in patient AM2, a normal finding for the patient's young age. Scale bar: 80 µm in A-B and 30 µm in A_1-B_1.

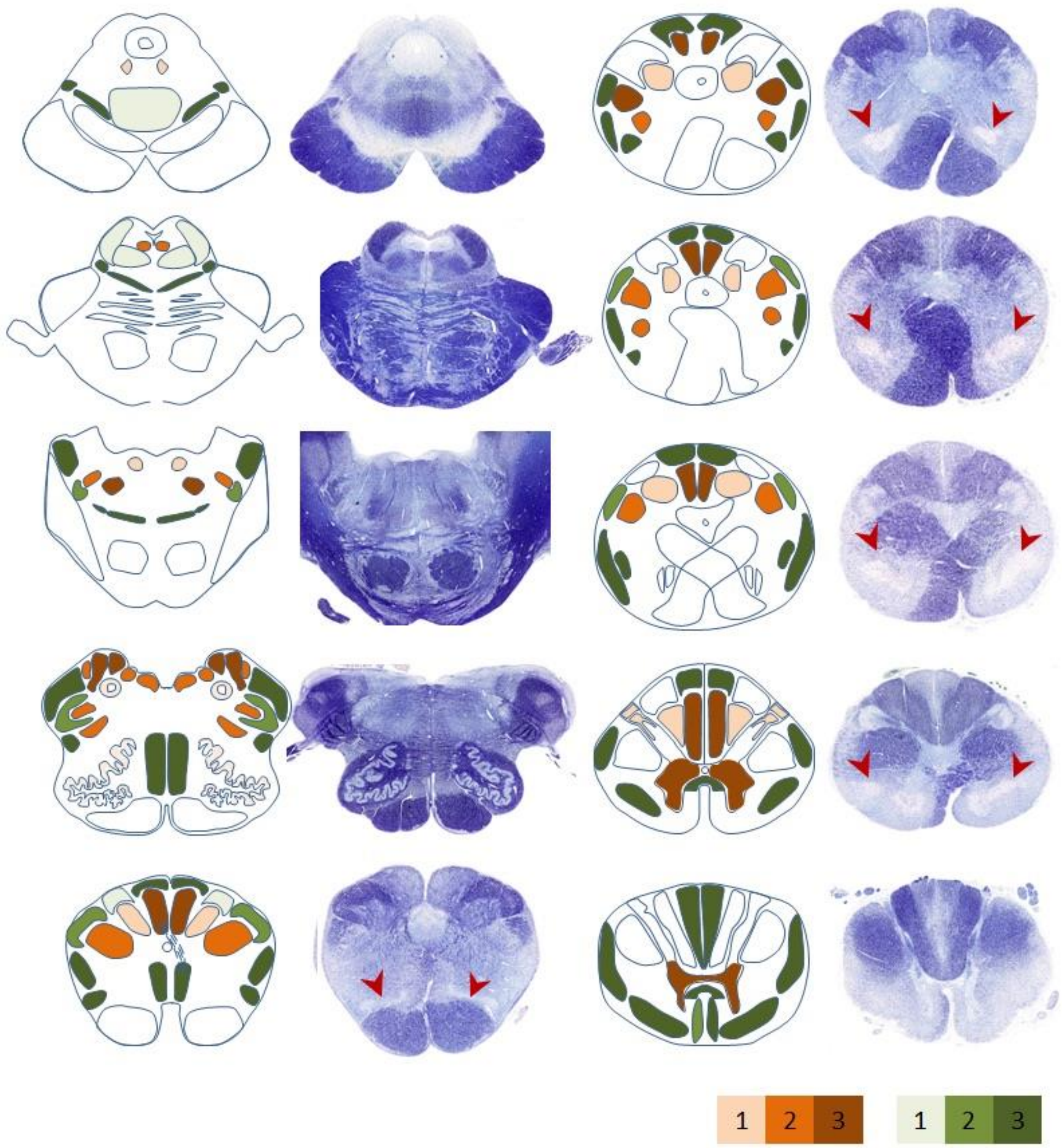

Figure S3. Schematic representation of the anatomical distribution and severity of the brain stem and spinal cord pathology in the patient AM4.

In the schematic figures the severity of grey matter pathology is indicated in orange with the lightest shade corresponding to mild neuronal atrophy and mild gliosis and the darkest shade corresponding to severe atrophy and gliosis. The severity of white matter tract pathology is indicated in green with the lightest shade corresponding to mild and the darkest shade corresponding to a severe fibre loss. The corresponding transverse brain stem and spinal cord sections are stained with luxol fast blue

special stain, where normally myelinated tracts are stained dark blue. The red arrowheads in the anterior aspect of the lower medulla and spinal cord indicate the bilateral symmetrical lesions.

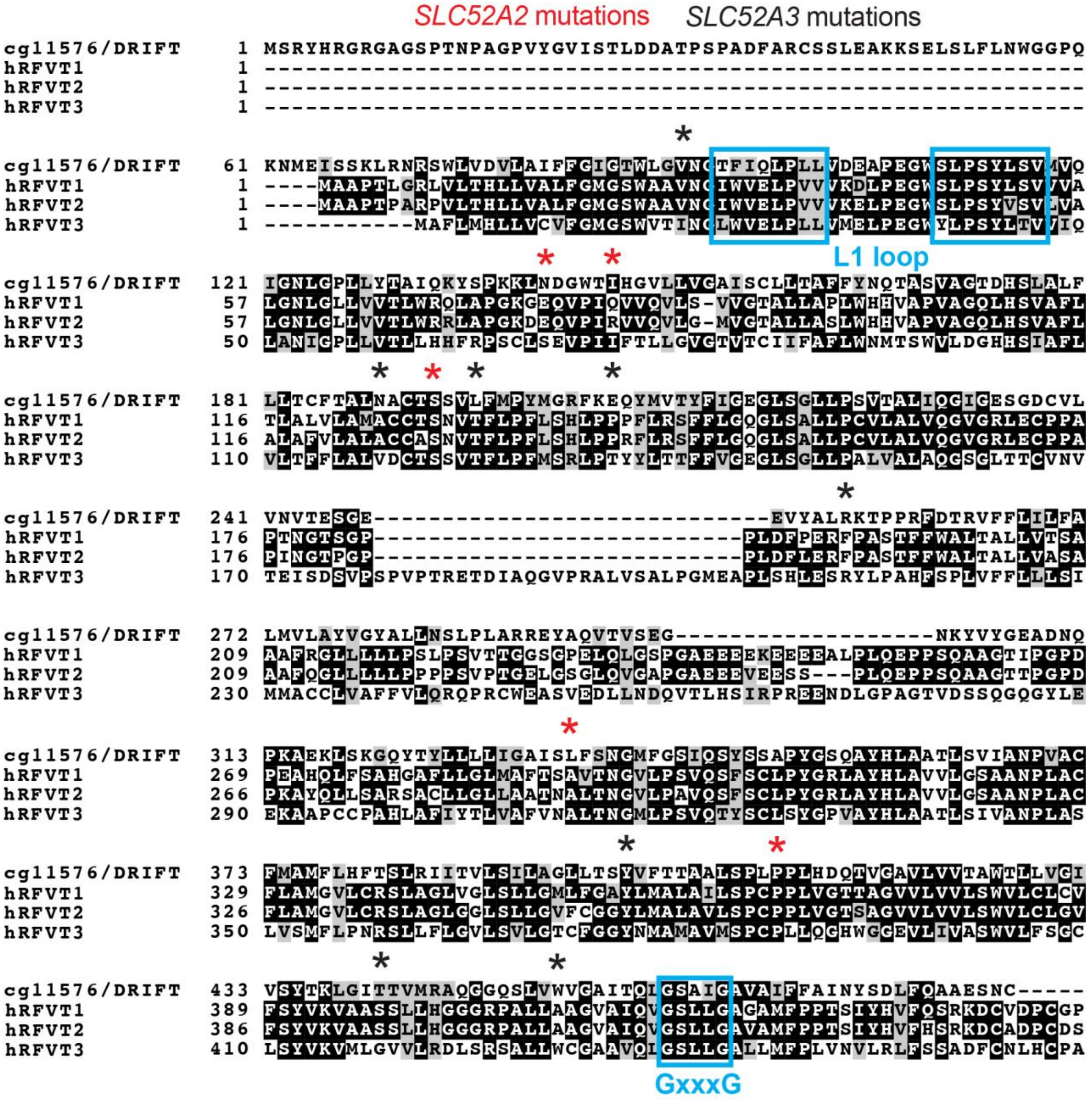

Figure S4. Structural conservation of amino acids between DRIFT, hRFVT1, hRFVT2 and hRFVT3. Novel mutations reported in this study are represented in red for *SLC52A2* and black for *SLC52A3*. Black: identical amino acid, Grey: functionally similar amino acids. The L1 loops (blue) and GXXXG motifs (blue) characteristic of RFVTs are shown. Conservation among species of the amino acid residues was determined using ClustalW2 software for multiple sequence alignment and plotted with BOXSHADE.

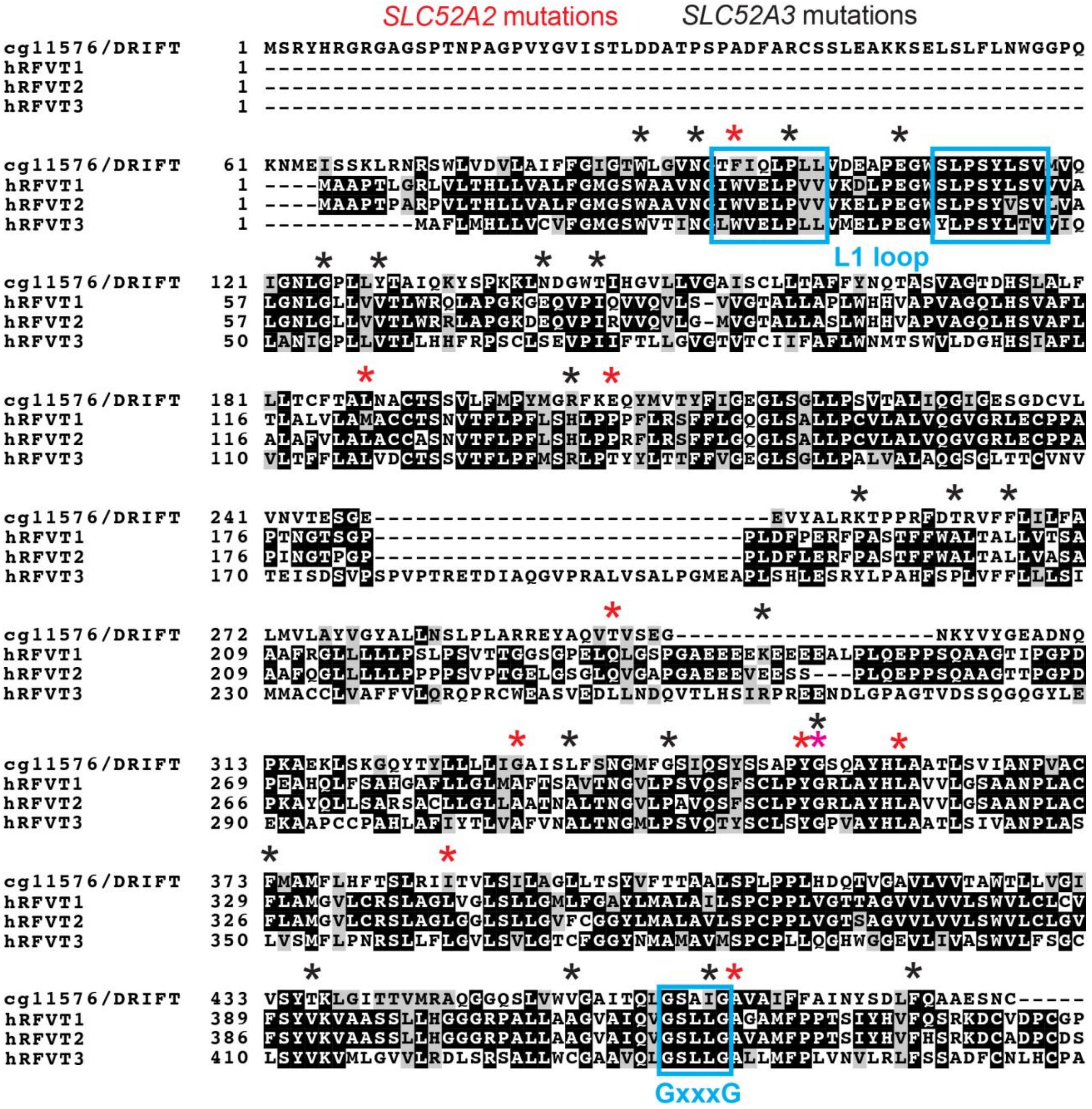

Figure S5. Conservation of previously described mutations in human RFVTs. Known mutations previously reported (Manole and Houlden, 2015) are represented in red for *SLC52A2* and black for *SLC52A3*. Additional annotations are described in Figure S4.

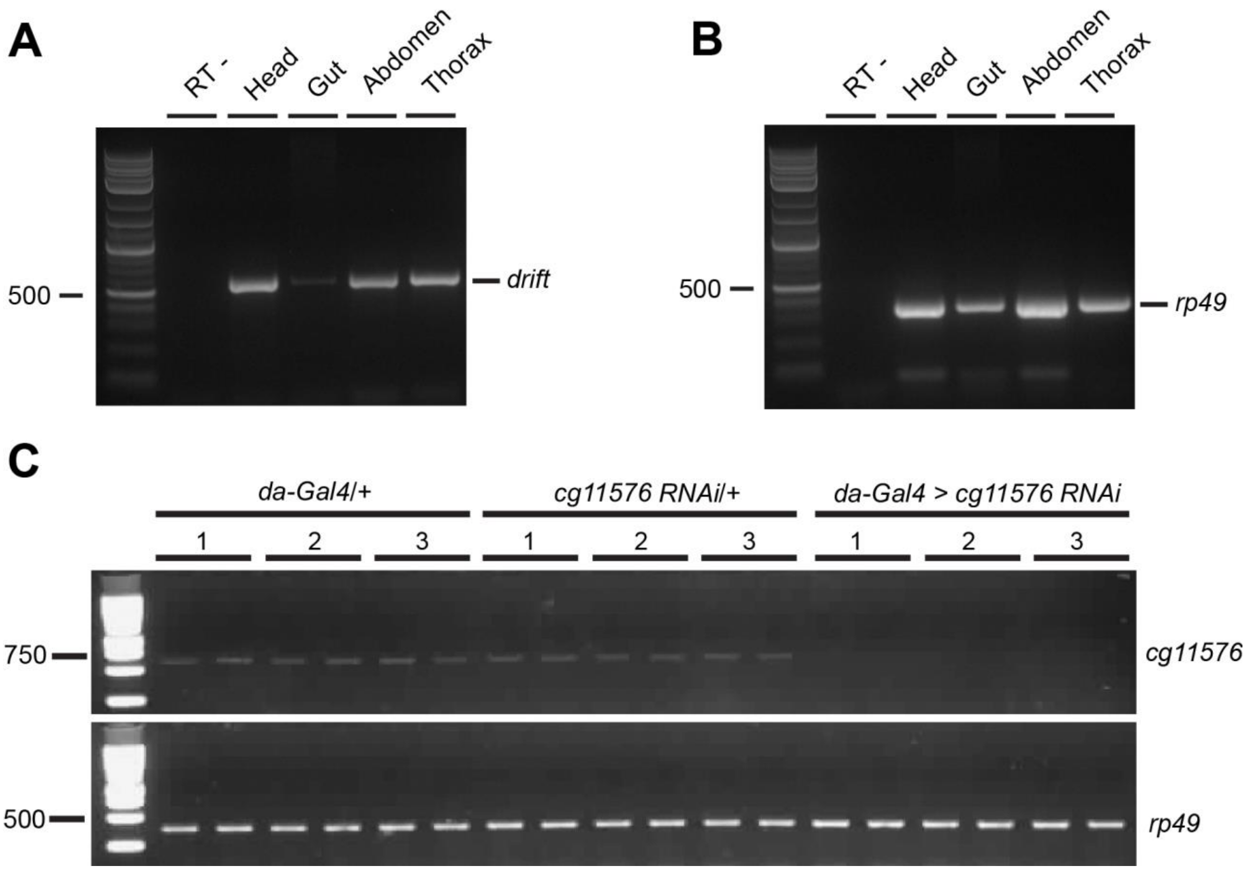

Figure S6. Knockdown of the *Drosophila* RFVT homologue *drift*.

(A, B) Expression of *drift* in head, gut, abdomen and thorax of the adult fly, detected through RT-PCR. The expected size of the bands are ~ 620 bp and ~ 370 bp for *drif*t (A) and *rp49* loading control (B) respectively. DNA ladder (left) is in bp. (C) Semi-quantitative RT-PCR illustrating *drift* knockdown by transgenic RNAi expressed using the global driver *daughterless*-GAL4 (*da*). *da* > + and + > *drift* RNAi are the controls for the driver and RNAi line respectively while the knockdown is represented by da > *drift* RNAi. Data were generated from three independent biological samples (1-3), each with two technical replicates.

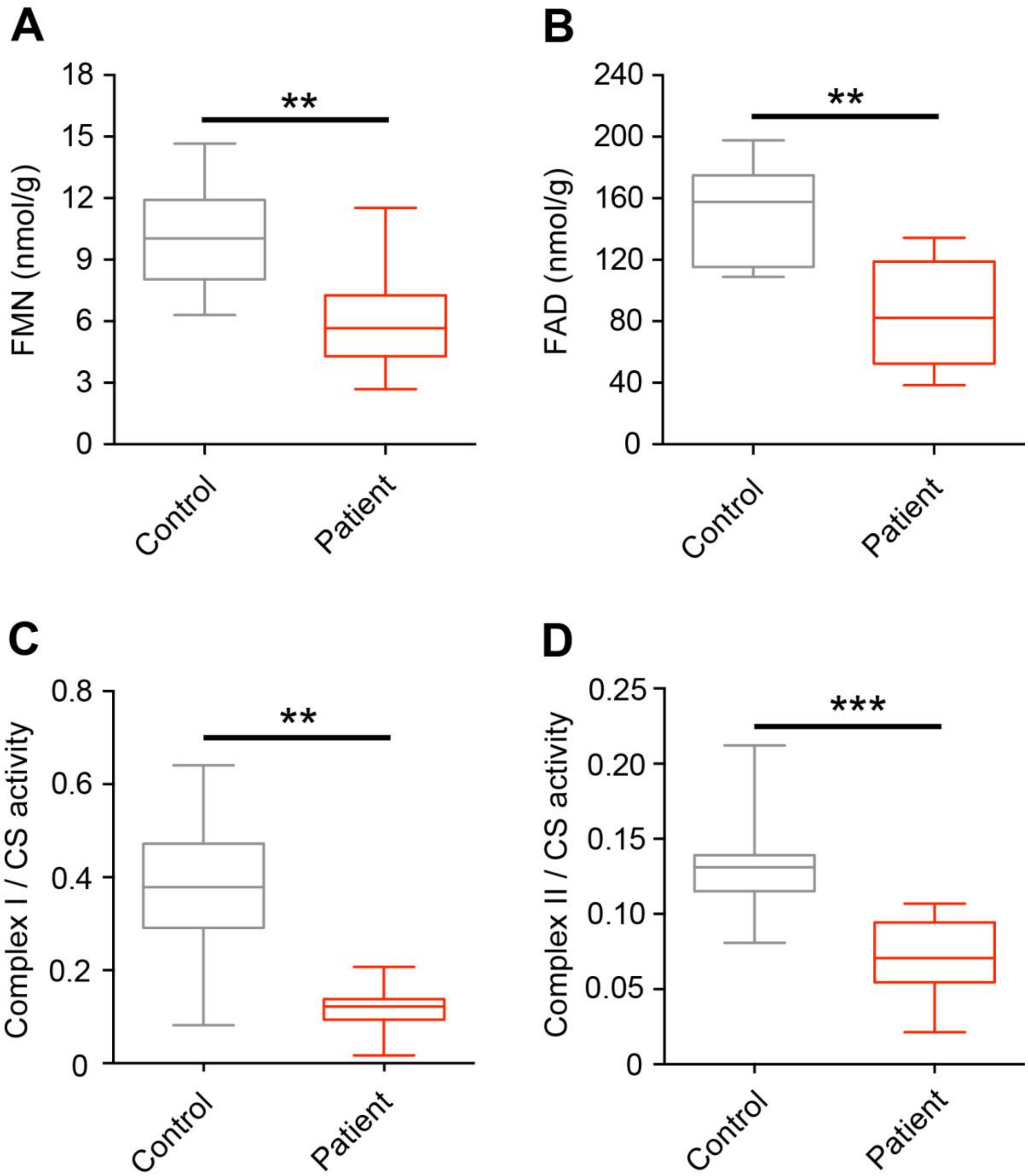

Figure S7. Reduced mitochondrial activity in BVVL syndrome patient fibroblasts.

(A-B) Intracellular FMN (A) and FAD (B) levels in control and patient fibroblasts. (C-D) Complex I (C) and complex II (D) activity in control and patients' fibroblasts. Complex activities are expressed as a ratio to citrate synthase activity. $**p < 0.005$, $***p < 0.0005$, Mann–Whitney U-test. Data were generated from a minimum of three independent experiments. n = 3 and n = 5 for control and patient fibroblasts respectively.

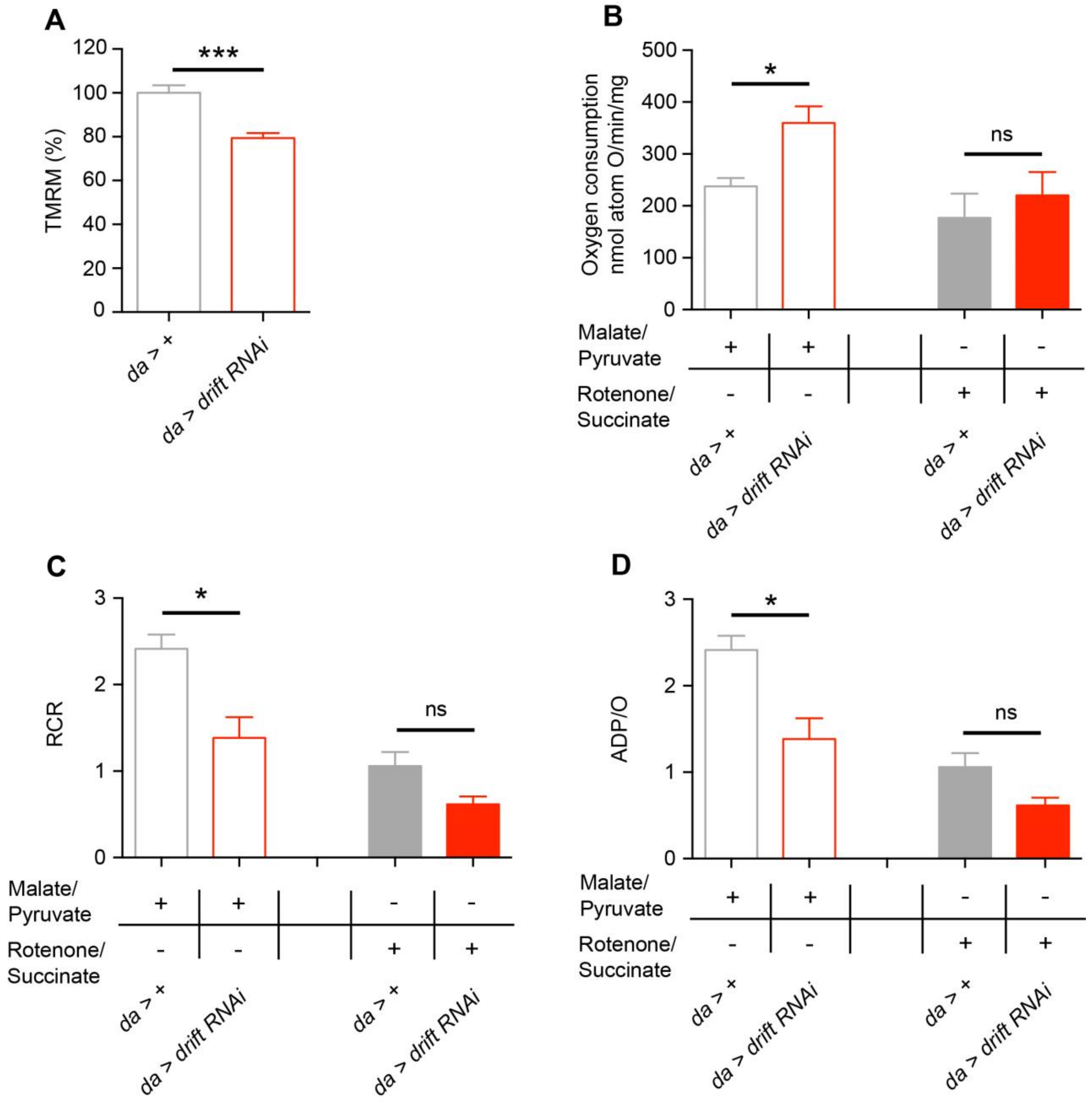

Figure S8. Knockdown of the *Drosophila* RFVT homologue *drift* results in reduced mitochondrial membrane potential and abnormal mitochondrial respiratory chain activity

(A) The mitochondrial membrane potential measured using TMRM fluorescence was significantly reduced in *drift* knockdown flies compared to *da* > + controls. *da* > +: n = 52; *da* > drift RNAi: n = 70 measurements from three brains for each genotype. (B) The respiratory chain activity was measured by assessing oxygen consumption and demonstrated that mitochondria from *drift* knockdown flies have increased complex I-dependent respiration compared to control flies. (C) The respiratory control ratio was significantly lower in mitochondria of *drift* knockdown flies compared to control mitochondria. (D) ADP/O levels were significantly lower in *drift* knockdown mitochondria compared to control. Complex I (+ malate/pyruvate): n = 5 technical replicates from

three biological replicates; complex II (+ rotenone/succinate): n = 3 technical replicates from three biological replicates. *$p < 0.05$, ***$p < 0.0005$, ns – $p > 0.05$, Mann-Whitney U-test.

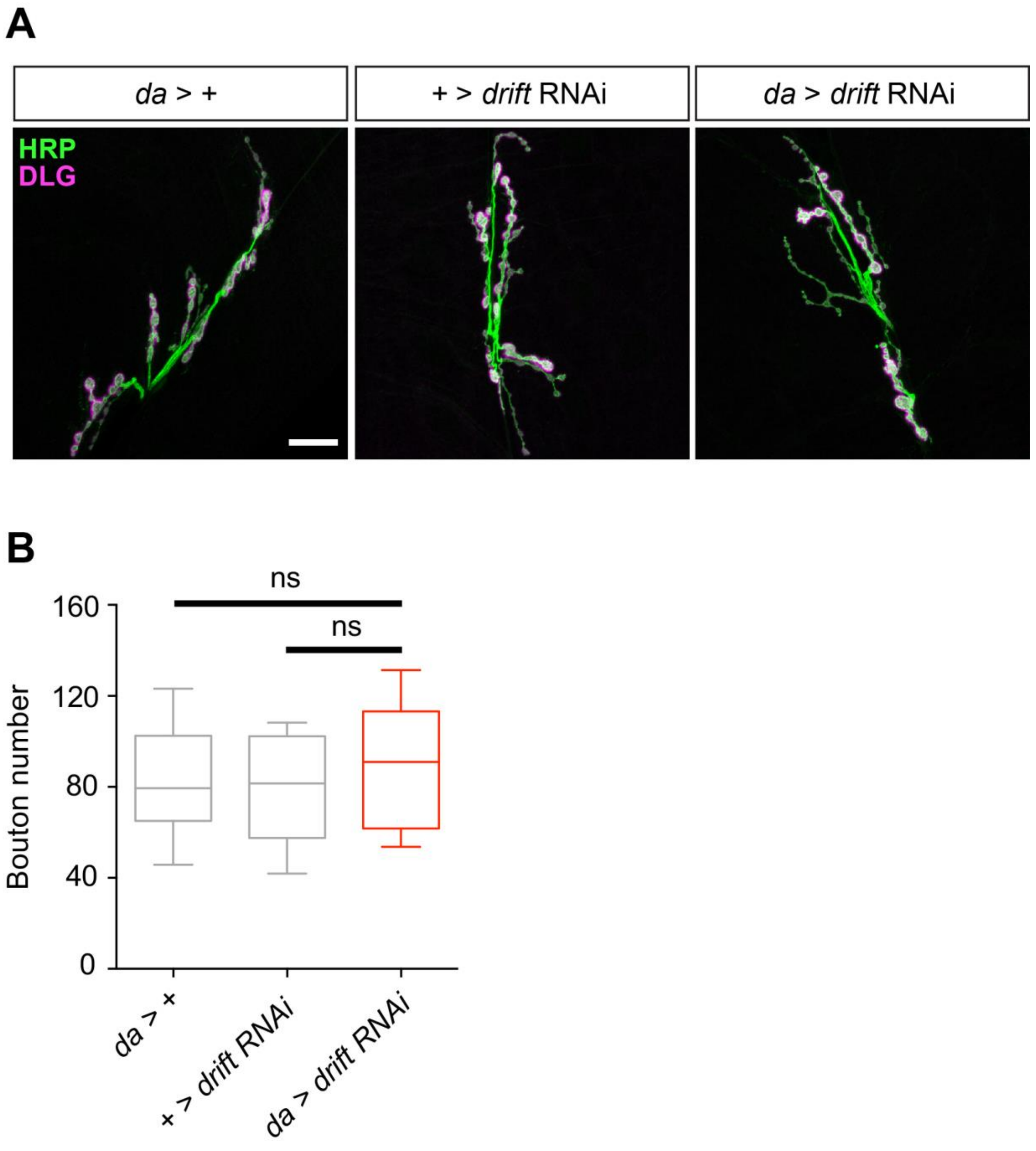

Figure S9. Motor neuron development is not grossly perturbed by *drift* knockdown. (A) Example confocal images of HRP-labelled 3rd instar larval motor neurons innervating muscle 6/7, segment 3. The post-synaptic sub-synaptic reticulem is labelled with anti-Discs Large (DLG). Scale bar: 20 µm. (B) Box plots showing median number of synaptic boutons in *drift* knockdown larvae and associated controls. *da* > +: n = 16; + > *drift* RNAi: n = 18; *da* > *drift* RNAi: n = 16. ns – p > 0.05, Kruskal-Wallis test with Dunn's post-hoc test.

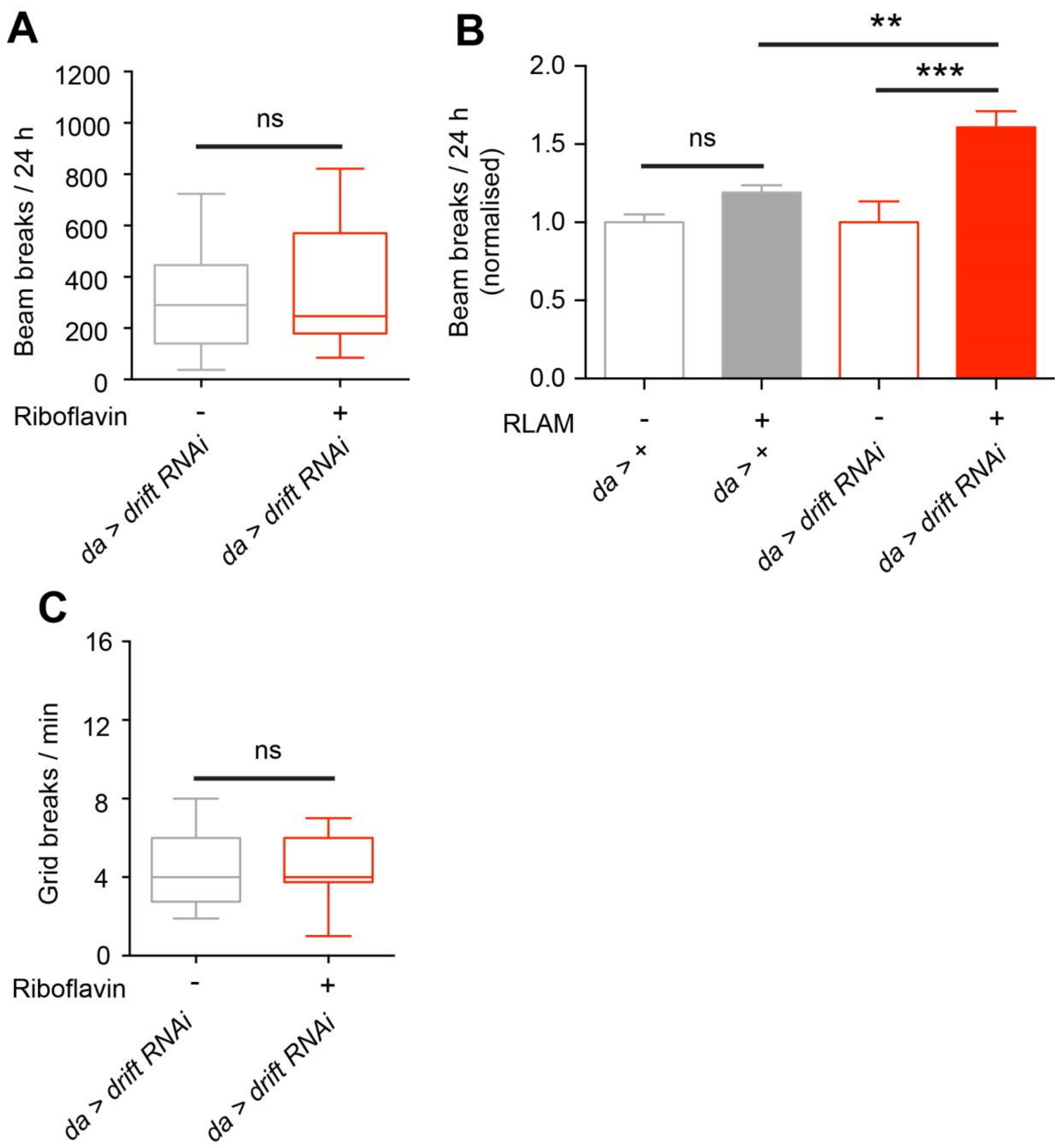

Figure S10. Riboflavin supplementation does not rescue locomotor defects in *drift* knockdown flies. (A) Total activity of *drift* knockdown adult female *Drosophila* over 24 h raised on normal or riboflavin-supplemented food. n = 15 and n =17 *drift* knockdown females respectively. (B) Normalised total activity over 24 h for *da* > + controls and *da* > *drift* RNAi adults given normal and RLAM-supplemented food. Data were generated from at least three independent experiments. n = 37-53. (C) Number of grid breaks per min by wandering 3rd instar larvae raised on normal or riboflavin-supplemented food. n = 30 for each genotype. **p < 0.005, ***p < 0.0005, ns – p > 0.05, Mann-Whitney U-test (A, C) or Kruskal-Wallis test with Dunn's post-hoc test (B).

Supplementary References

Birch-Machin, M.A., et al., 1994. An evaluation of the measurement of the activities of complexes I-IV in the respiratory chain of human skeletal muscle mitochondria. Biochem Med Metab Biol. 51**,** 35-42.

Ferguson, M., et al., 2005. Age-associated decline in mitochondrial respiration and electron transport in Drosophila melanogaster. Biochem J. 390**,** 501-11.

Giancaspero, T.A., et al., 2009. The occurrence of riboflavin kinase and FAD synthetase ensures FAD synthesis in tobacco mitochondria and maintenance of cellular redox status. Febs j. 276**,** 219-31.

Hargreaves, I.P., Heales, S.J., Land, J.M., 1999. Mitochondrial respiratory chain defects are not accompanied by an increase in the activities of lactate dehydrogenase or manganese superoxide dismutase in paediatric skeletal muscle biopsies. J Inherit Metab Dis. 22**,** 925-31.

Kinghorn, K.J., et al., 2015. Loss of PLA2G6 leads to elevated mitochondrial lipid peroxidation and mitochondrial dysfunction. Brain. 138**,** 1801-16.

Ragan, C.I., Wilson, M. T., Darley-Usmar, V.M, Lowe, P.N 1987. Subfractination of mitochondria and isolation of the proteins of oxidative phosphorylation. Mitochondria: a practical approach. IRL Press Limited, Oxford, England**,** 79-112.

Shephard, J.A.a.G., P.B., 1969. Citrate synthase from rat liver. Methods in Enzymology. 13**,** 11-19.
